# Cardiac-targeting peptide–modified Prussian blue nanozymes loaded with Astragaloside IV for efficient ICI-myocarditis therapy

**DOI:** 10.1016/j.mtbio.2026.103482

**Published:** 2026-07-22

**Authors:** Jing Zhou, Jiali Tang, Zehao Huang, Zixuan Liang, Yunxi Huang, Qi Zeng, Chao Yu, Junjie Liu, Yuanyuan Chen, Sida Wang, Nuo Yang, Yan Deng

**Affiliations:** a Department of Radiology, The First Affiliated Hospital of Guangxi Medical University, No. 6, Shuangyong Road, Nanning, 530021, China; b Department of Ultrasound, The First Affiliated Hospital of Guangxi Medical University, No. 6, Shuangyong Road, Nanning, 530021, China; c Department of Ultrasound, Guangxi Medical University Cancer Hospital, No.71 Hedi Road, Nanning, 530021, China; d Department of Cardiothoracic Surgery, Cardiovascular Institute, The First Affiliated Hospital of Guangxi Medical University, No. 6, Shuangyong Road, Nanning, 530021, China; e Department of Otolaryngology-Head and Neck Surgery, Guangxi Medical University Cancer Hospital, No.71 Hedi Road, Nanning, 530021, China; f Colorectal and Anal Disease Unit, Department of Gastrointestinal Surgery, Guangxi Medical University Cancer Hospital, No.71 Hedi Road, Nanning, 530021, China

**Keywords:** Prussian blue nanozyme, Immunoredox regulation, Macrophage polarization, Immune checkpoint inhibitor–associated myocarditis

## Abstract

Immune checkpoint inhibitor (ICI) therapy can trigger immune-related adverse events across multiple organs, and myocarditis remains among the most lethal cardiac manifestations, culminating in arrhythmias and heart failure. Current management primarily relies on ICI interruption and empirical immunosuppression, but this broad strategy lacks heart-targeted precision and does not specifically address the concurrent immune and oxidative components of myocardial injury. Two major challenges therefore remain: insufficient therapeutic exposure within inflamed myocardium and the need to restrain pathogenic immune activation while protecting stressed cardiomyocytes from oxidative damage. Astragaloside IV (AS-IV) is appealing for pleiotropic cardioprotection and immunoregulation, yet limited bioavailability and insufficient myocardial exposure constrain its in vivo efficacy. Here we develop AS@PB-CTP, a cardiac-homing nanozyme integrating Prussian blue (PB) with SOD/CAT/POD-like catalysis, an AS-IV payload, and a cardiac-targeting peptide (CTP). AS@PB-CTP attenuates oxidative stress–induced injury in H_2_O_2_-challenged HL-1 cardiomyocytes and modulated macrophage-associated phenotypes toward an M2-like state. In the anti-PD-1/cTnI myocarditis model, AS@PB-CTP preferentially accumulates in injured myocardium, reduces leukocyte infiltration and fibrosis, and improves ventricular function, with increased ejection fraction and fractional shortening. Mechanistically, transcriptomic and protein-level analyses identified PI3K–Akt–mTOR signaling as a treatment-associated pathway, while immune profiling revealed coordinated attenuation of splenic IFN-γ^+^CD8^+^ effector T-cell responses and cardiac macrophage-associated inflammation. Collectively, AS@PB-CTP represents a cardiac-targeted redox–immune nanotherapeutic platform that couples catalytic antioxidant activity with immune modulation for ICI-associated myocarditis.

## Highlights

• AS@PB-CTP disrupts the inflammation–oxidative stress loop in ICI-myocarditis through its dual-action design: a PB nanozyme core for ROS scavenging and an AS-IV coating for immunomodulation.
• AS@PB-CTP achieves cardiac-targeted delivery to inflamed myocardium via CTP surface design, enhancing therapeutic precision while reducing systemic off-target exposure.
• AS@PB-CTP provides direct cardiomyocyte protection through anti-apoptosis and translates it into quantified functional benefit in vivo, evidenced by improved EF/FS and reduced myocardial damage.
• By modulating the PI3K–Akt axis, AS@PB-CTP bridges its material action to cardiac functional recovery, providing a comparable and interpretable mechanistic explanation.
• AS@PB-CTP promotes macrophage polarization toward reparative phenotypes, thereby reprogramming the inflammatory milieu to mitigate ongoing myocardial injury and fibrosis.

## Graphical abstract

**Figure:**
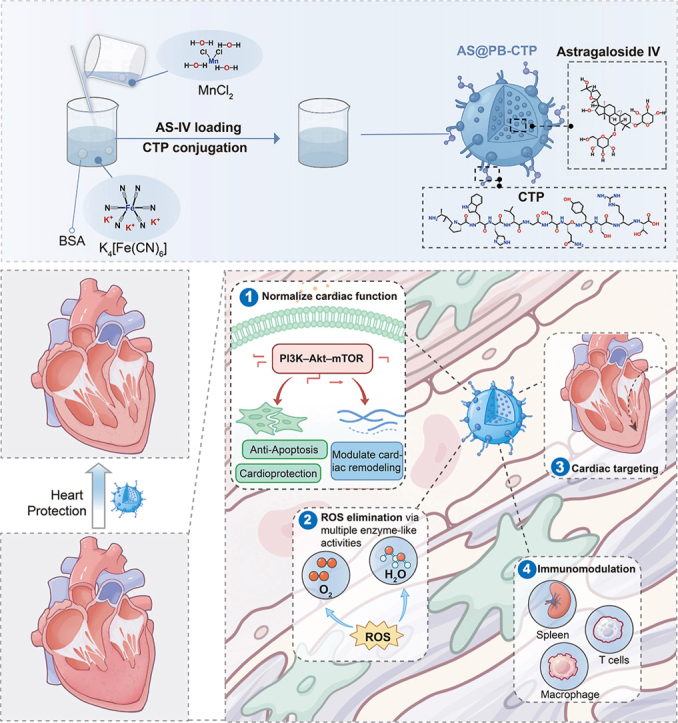

## 1 Introduction

Immune checkpoint inhibitors (ICIs), especially monoclonal antibodies targeting CTLA-4, PD-1, and PD-L1, have transformed the treatment of advanced malignancies by revitalizing antitumor T-cell immunity [[1], [2], [3]]. Since their clinical introduction in 2011, ICIs have significantly enhanced long-term survival across various cancer types. Nonetheless, this therapeutic progress is accompanied by an expanding range of immune-related adverse events (irAEs) resulting from immune hyperactivation [3]. Among these complications, ICI-associated myocarditis (ICI-MC) is uncommon, with a reported incidence generally below 2%, but is associated with disproportionately high mortality and a substantial risk of arrhythmia, conduction disturbance, heart failure, and major adverse cardiovascular events [4]. This life-threatening cardiotoxicity may limit the overall clinical benefit of ICI therapy and underscores the urgent need for strategies that protect the heart during cancer immunotherapy [5].

At the mechanistic level, ICI-MC results from the disruption of key immune checkpoints, particularly the PD-1/PD-L1 axis, which maintains cardiac immune tolerance [6]. PD-1 is expressed on T cells, NK cells, B cells, and monocytes, and it interacts with PD-L1 and PD-L2 on antigen-presenting cells, as well as on non-immune cells such as cardiomyocytes and endothelial cells. Blocking this axis removes critical inhibitory signals, allowing autoreactive cytotoxic T cells to infiltrate the myocardium, recognize cardiac antigens, and cause sustained tissue damage. This process activates the IFN-γ–STAT1 pathway and the CXCL9/CXCL10–CXCR3 chemokine loop, thereby further amplifying T-cell recruitment and sustaining inflammation [7].

Recent evidence further indicates that local interactions among cardiac macrophages, T cells, fibroblasts, and other resident cells contribute to the recruitment and maintenance of pathogenic immune populations within the myocardium [8]**.** Concurrently, activated immune cells and damaged cardiomyocytes produce excessive reactive oxygen species (ROS), leading to mitochondrial dysfunction, lipid peroxidation, and inflammatory cardiomyocyte death. This cycle of immune hyperactivation and redox imbalance is the core pathological feature of ICI-MC and poses a significant challenge to effective intervention [9,10].

Current clinical management primarily relies on high-dose corticosteroids. However, an increasing number of patients exhibit steroid resistance, resulting in persistent inflammation and a significantly elevated risk of major adverse cardiovascular events (MACEs) [11]. Moreover, conventional immunosuppressive therapies broadly inhibit T-cell activation and inflammatory signaling and may therefore interfere with the antitumor immune responses required for ICI efficacy. No targeted therapies have been approved to directly disrupt the underlying pathogenic immune pathways. Consequently, there is an urgent need to develop and implement therapeutic strategies that attenuate pathological cardiac inflammation and downstream tissue injury while minimizing generalized systemic immunosuppression [[11], [12], [13]].

Given the intertwined nature of immunological and oxidative pathologies, an ideal therapeutic strategy should simultaneously attenuate excessive inflammatory signaling, effectively scavenge ROS, protect mitochondria, and preferentially accumulate in inflamed myocardial tissue. Existing pharmacological approaches rarely combine these complementary functions. Traditional immunosuppressants act systemically and fail to modulate redox homeostasis, whereas antioxidants are hindered by poor stability, low bioavailability, and minimal cardiac targeting. This significant therapeutic gap underscores the need for multifunctional nanoplatforms that can deliver myocardial treatment and achieve dual immune–redox modulation [14].

**Fig. 1:**
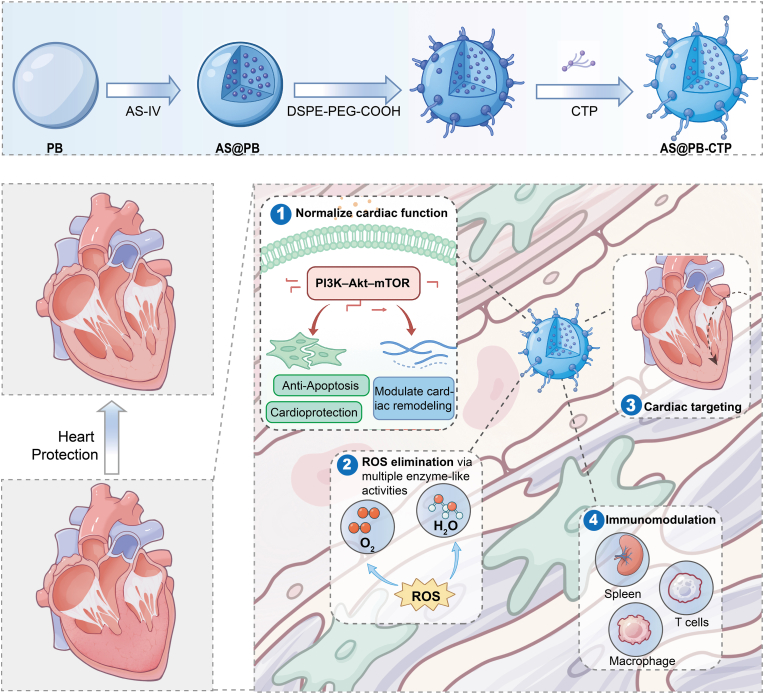
**Schematic illustration of AS@PB-CTP fabrication and its cardioprotective mechanisms.** The multifunctional nanozyme AS@PB-CTP was constructed through a sequential assembly process: AS-IV was first loaded into Prussian Blue (PB) nanozyme cores to form AS@PB, followed by surface coating with DSPE-PEG-COOH and conjugation of the CTP to yield the final AS@PB-CTP construct (top panel). Upon systemic administration, CTP directs the preferential accumulation of AS@PB-CTP within injured myocardial tissue. At the target site, AS@PB-CTP exhibits multi-enzyme mimetic activity, catalytically scavenging excess ROS and alleviating oxidative stress. In parallel, it modulates the PI3K–Akt–mTOR signaling pathway, reducing cardiomyocyte apoptosis, enhancing cardiac function, and attenuating adverse remodeling. Moreover, AS@PB-CTP actively regulates immune cells—including macrophages and T cells—in both splenic and cardiac compartments, thereby contributing to comprehensive cardioprotection. (For interpretation of the references to color in this figure legend, the reader is referred to the Web version of this article.)

To tackle these problems, we designed a multifunctional nanozyme called AS@PB-CTP. This system aims to act on both the immune and oxidative components of ICI-MC and to target injured heart tissue (Fig. 1). Prussian blue (PB) nanoparticles form the core of the system. PB has superoxide dismutase- and catalase-like activities. These properties allow PB to continuously clear superoxide and hydrogen peroxide, restore redox balance, and help protect mitochondria in inflamed heart tissue [15,16]. Astragaloside IV (AS-IV), a natural saponin from *Astragalus membranaceus*, works together with PB. AS-IV helps reduce pro-inflammatory cytokines and has been associated with modulation of inflammation-, survival-, and remodeling-related signaling pathways, including the PI3K–Akt–mTOR axis [[17], [18], [19]]. A cardiac-targeting peptide (CTP) is attached to the PEGylated PB surface. CTP helps AS@PB-CTP home to diseased heart tissue and increases local drug levels [20,21]. These three parts work together as one system. AS@PB-CTP delivers both enzymatic antioxidant activity and immune-regulating signals to the inflamed myocardium. In our ICI-MC model, AS@PB-CTP was associated with improved redox homeostasis, reduced pathological PI3K–Akt–mTOR signaling, attenuation of inflammatory immune activation, and modulation of cardiac macrophage phenotypes toward a reparative-like state [22]**.** In this way, the nanozyme limits oxidative damage and calms abnormal immune responses. As a result, AS@PB-CTP effectively reduces immune-mediated injury to the heart.

## 2 Results and discussion

### 2.1 Rational design, self-assembly, and physicochemical characterization of the AS@PB-CTP nanozyme

**Fig. 2:**
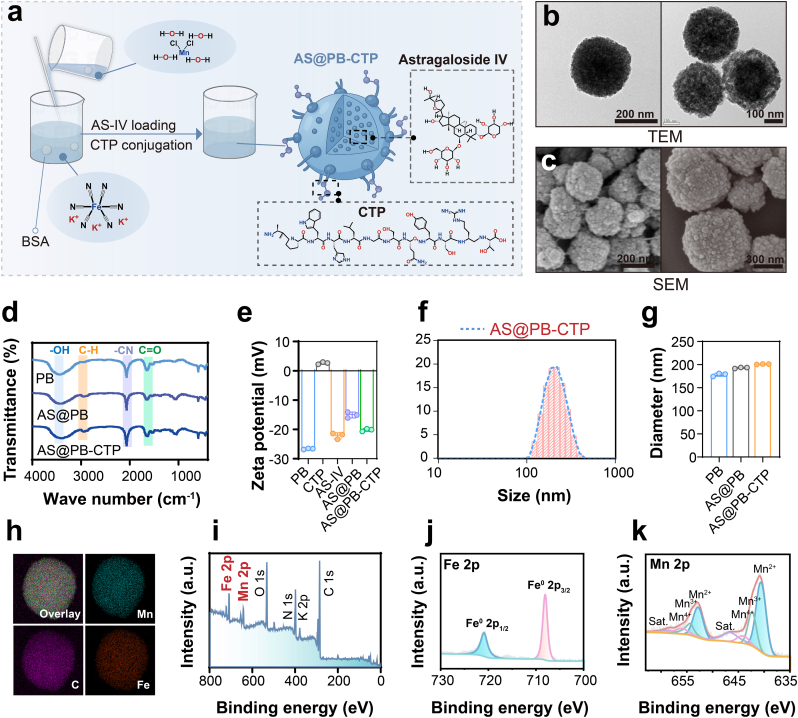
**Schematic illustration, morphology, and physicochemical characterization of AS@PB-CTP.** (a) Schematic of the BSA-assisted co-assembly of AS-IV and a cardiomyocyte-targeting peptide (CTP) with Prussian blue (PB) to form the AS@PB-CTP nanozyme. (b, c) TEM and SEM images showing uniformly dispersed, quasi-spherical AS@PB-CTP nanoparticles with rough surfaces. (d) FT-IR spectra of PB, AS@PB, and AS@PB-CTP. The characteristic –CN stretching band of the PB framework is preserved, while additional C–H and C=O/amide (–CO–NH–) bands appear and are enhanced after AS-IV loading and CTP conjugation, confirming successful surface functionalization. (e) Zeta potentials of PB, CTP, AS-IV, AS@PB and AS@PB-CTP, demonstrating stepwise surface charge shifts upon sequential AS-IV incorporation and CTP modification. (f) DLS size-distribution profile of AS@PB-CTP, indicating a narrow monomodal hydrodynamic size distribution. (g) Comparison of average hydrodynamic diameters of PB, AS@PB, and AS@PB-CTP. (h) STEM–EDS elemental mapping of a representative nanoparticle showing homogeneous distribution of Mn, Fe, and C. (i) XPS survey spectrum of AS@PB-CTP. (j,k) High-resolution Fe 2p and Mn 2p XPS spectra, confirming the coexistence of Fe(II)/Fe(III) and Mn(II)/Mn(III) redox couples within the nanozyme framework. (For interpretation of the references to color in this figure legend, the reader is referred to the Web version of this article.)

AS@PB-CTP nanozymes were prepared via an albumin-assisted, stepwise assembly strategy (Fig. 2a) [23]. PBnanoparticles were first synthesized in the presence of bovine serum albumin (BSA) to obtain BSA-stabilized PB cores. AS-IV was then loaded onto the PB@BSA nanoparticles, followed by insertion of DSPE–PEG(2000)–COOH and subsequent covalent conjugation of the CTP to the PEG corona via EDC/NHS chemistry. Transmission electron microscopy (TEM) and scanning electron microscopy (SEM) images clearly showed that the obtained AS@PB-CTP nanoparticles possessed a uniform near-spherical morphology with a rough, textured surface that is favorable for drug loading and catalytic reactions (Fig. 2b and c). Fourier-transform infrared (FT-IR) spectra of PB, AS@PB and AS@PB-CTP exhibited a broad band at 3200–3600 cm^−1^ and several bands at 2850–2950 cm^−1^, which can be assigned to O–H and aliphatic C–H stretching vibrations originating from surface-bound water and organic components (Fig. 2d). All three samples showed a characteristic C ≡ N stretching band of the PB framework at around 2080 cm^−1^, indicating that the PB lattice is preserved after modification. In contrast, the carbonyl/amide (C=O, –CO–NH–) band near 1630 cm^−1^ became markedly more intense for AS@PB and AS@PB-CTP than for bare PB, supporting the successful incorporation of BSA-, AS-IV-, and CTP-associated organic components. The X-ray diffraction (XRD) pattern of AS@PB-CTP (Fig. S1a) displayed the characteristic diffraction peaks of PB and showed no new peaks, further confirming that the crystalline PB framework remained intact after AS-IV loading and surface functionalization. The zeta potential changed stepwise from PB to AS@PB and finally to AS@PB-CTP (Fig. 2e), indicating progressive ligand modification on the PB surface [24]. Dynamic light scattering (DLS) analysis revealed a narrow hydrodynamic size distribution for AS@PB-CTP (Fig. 2f), and comparison of the average diameters of PB, AS@PB and AS@PB-CTP (Fig. 2g) demonstrated that surface engineering only slightly increased particle size while maintaining good colloidal stability. AS@PB-CTP exhibited a uniform hydrodynamic diameter of 200.7±0.53 nm with a low polydispersity index (PDI = 0.072±0.019), indicative of excellent dispersion stability (Supplementary Table S1). To further evaluate its colloidal stability under physiologically relevant conditions, AS@PB-CTP was incubated in phosphate-buffered saline (PBS) and Dulbecco's modified Eagle medium (DMEM) supplemented with 10% fetal bovine serum (FBS) at 37 °C for 48 h. Its hydrodynamic diameter and zeta potential remained stable throughout the observation period, with no visible aggregation or precipitation, demonstrating favorable colloidal stability under physiological and serum-containing conditions (Fig. S1b–d). Elemental mapping confirmed that Mn, Fe, and C were evenly distributed within each nanoparticle (Fig. 2h). X-ray photoelectron spectroscopy (XPS) survey scans showed clear signals for Fe, Mn, N, C, and K (Fig. 2i), suggesting that these elements were successfully introduced into the AS@PB-CTP structure. High-resolution spectra of Fe 2p and Mn 2p further revealed the presence of both Fe(II)/Fe(III) and Mn(II)/Mn(III) oxidation states (Fig. 2j and k). These redox pairs reflect the nanozyme's multi-enzyme-like catalytic potential. Drug loading studies showed that AS had a loading efficiency (LE%) of 5.37 ± 0.21% and an encapsulation efficiency (EE%) of 63.4 ± 1.05% (Supplementary Table S1**)**, indicating that the system successfully incorporated the drug. In vitro release experiments were conducted in PBS with 0.5% polysorbate 80. The release profile showed a biphasic pattern, with an initial rapid-release phase followed by a slower sustained-release phase. By 24 h, cumulative release reached 87.20 ± 1.78% (Fig. S2). The amount of CTP associated with AS@PB-CTP after EDC/NHS-mediated coupling and purification was determined to be 162.0 ± 7.9 μg mg^−1^ AS@PB-CTP, corresponding to a CTP content of 16.20 ± 0.79 wt% in the final formulation **(**Supplementary Table S2**)**. This result suggests that AS@PB-CTP provides both rapid drug availability and extended exposure, which may benefit therapeutic outcomes.

### 2.2 Network pharmacology-guided target identification and molecular docking of AS-IV

**Fig. 3:**
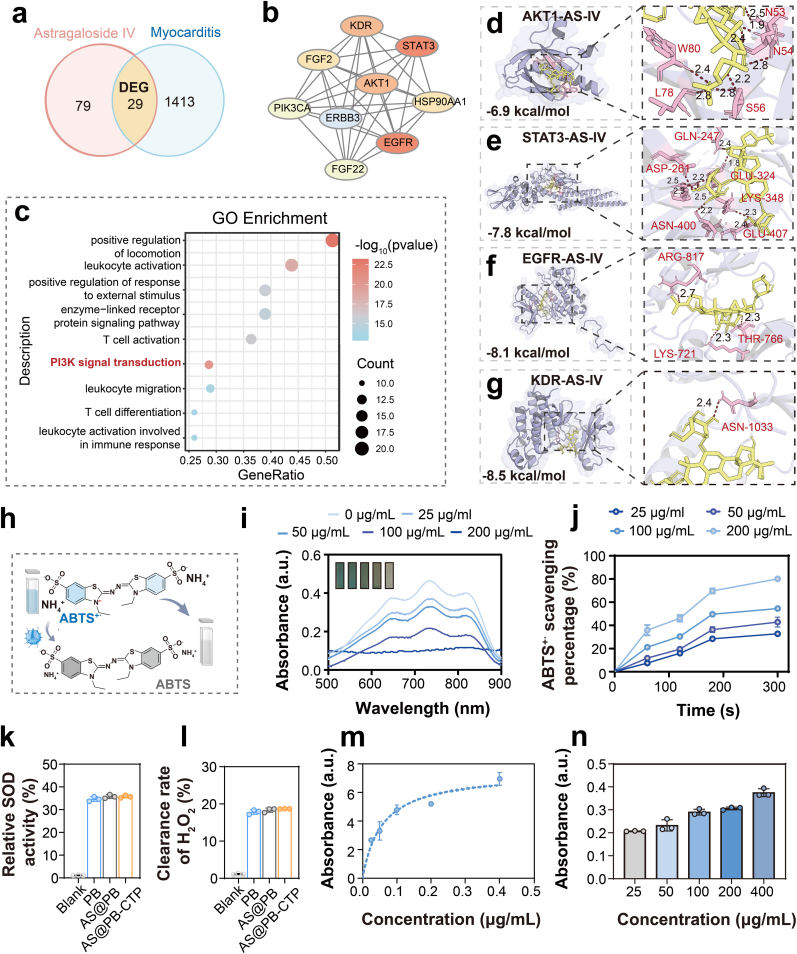
**Network-based target identification, molecular docking, and enzyme-mimetic antioxidant activities of AS@PB-CTP.** (a) Venn diagram showing 29 intersecting genes between AS-IV–related and myocarditis-related gene sets. (b) Protein–protein interaction (PPI) network of overlapping targets related to AS-IV and myocarditis, highlighting AKT1, STAT3, EGFR, and KDR as central hubs. (c) Gene Ontology (GO) enrichment of shared genes indicating involvement in PI3K signalling, T-cell activation, and immune-regulatory pathways. (d–g) Molecular docking of AS-IV with AKT1, STAT3, EGFR, and KDR; representative binding poses with magnified hydrogen-bond/interaction details are shown. (h) Schematic of the ABTS^•+^ radical-scavenging assay used to evaluate the total antioxidant capacity of AS@PB-CTP. (i,j) UV–vis spectra and time-dependent ABTS^•+^ scavenging by AS@PB-CTP at varying concentrations. (k) SOD-like activities of PB, AS@PB, and AS@PB-CTP, determined by inhibition of superoxide-dependent formazan formation. The reaction system without nanozyme served as the negative control. (l) H_2_O_2_ clearance by PB, AS@PB, and AS@PB-CTP in the CAT-like assay. The H_2_O_2_-containing reaction without nanozyme served as the negative control. (m) POD-like catalytic behaviour exhibiting apparent saturation kinetics (Michaelis–Menten fit shown). (n) Concentration-dependent POD-like activity of AS@PB-CTP.

To explore the potential molecular targets underlying the cardioprotective effects of AS-IV, we used a network pharmacology approach. We first predicted and reported targets of AS-IV and compared them with myocarditis-related genes. This comparison yielded 29 overlapping candidate targets (Fig. 3a).

We then built a protein–protein interaction (PPI) network using these shared genes (Fig. 3b). Among the nodes, AKT1, STAT3, EGFR, and KDR were identified as potential hub targets, suggesting that they may participate in the pharmacological actions of AS-IV. Gene Ontology (GO) enrichment analysis showed that the DEGs were strongly involved in immune-related processes such as T cell activation, leukocyte migration, and cytokine signaling. Importantly, these genes were also enriched in the PI3K–Akt pathway, which is involved in immune regulation, cellular survival, metabolism, and oxidative-stress responses (Fig. 3c). To further evaluate the potential interactions between AS-IV and the identified hub targets, molecular docking studies were performed. AS-IV showed favorable predicted binding affinities toward AKT1, STAT3, EGFR, and KDR (Fig. 3d–g). The compound formed predicted hydrogen bonds with key residues such as GLN247 and ASN1033. These results indicate that AS-IV has the potential to interact with and modulate kinase activity and downstream inflammatory signaling. This structural evidence supports the inclusion of AS-IV in the AS@PB-CTP platform as a rational component for targeted immunomodulatory therapy.

### 2.3 Enzyme-mimetic antioxidant activity and kinetic behaviour of AS@PB-CTP

To systematically characterize the radical-scavenging and enzyme-mimetic properties of AS@PB-CTP, we initially employed the ABTS assay under aqueous conditions. ABTS^•+^ radicals, generated via oxidation of ABTS, exhibit a characteristic UV–vis absorption peak at 734 nm (Fig. 3h). Upon incubation with increasing concentrations of AS@PB-CTP (25–200 μg/mL), the intensity of this peak progressively decreased (Fig. 3i), indicating efficient ABTS^•+^ radical scavenging in a concentration-dependent manner. Correspondingly, the ABTS^•+^ scavenging percentage increased with both nanozyme concentration and reaction time, reaching its highest level at 200 μg/mL within 300 s (Fig. 3j), demonstrating rapid and concentration-dependent radical-neutralizing activity [25]. To further validate the ROS-scavenging capacity of AS@PB-CTP, a DPPH radical-scavenging assay was performed at physiological pH 7.4 and mildly acidic pH 6.5. AS@PB-CTP induced a concentration-dependent decrease in the characteristic DPPH absorbance at 517 nm and maintained effective radical-scavenging activity under both pH conditions. These results provide complementary colorimetric evidence for the antioxidant capacity of AS@PB-CTP under physiological and mildly acidic conditions (Fig. S3a–c).

To dissect the specific enzyme-mimetic components underlying this antioxidant activity, we next assessed the individual catalytic functions. AS@PB-CTP exhibited pronounced superoxide dismutase (SOD)-like activity, comparable to that of PB and AS@PB, as evidenced by the inhibition of superoxide-induced formazan formation relative to the corresponding blank control (Fig. 3k). Additionally, AS@PB-CTP showed a high H_2_O_2_ clearance rate in a catalase (CAT)-like assay compared with the H_2_O_2_-containing control without nanozyme (Fig. 3l), consistent with catalase-like decomposition of H_2_O_2_. To elucidate the peroxidase (POD)-like mechanism, we performed steady-state kinetic analysis by varying TMB concentrations at a fixed nanozyme dose. The reaction followed typical Michaelis–Menten saturation kinetics (Fig. 3m), and the reaction rate, reflected by absorbance changes, increased proportionally with AS@PB-CTP concentration (Fig. 3n), supporting its concentration-dependent POD-like catalytic activity [26]. The POD-like assay was used to characterize the catalytic versatility of AS@PB-CTP, whereas its net antioxidant behavior was independently supported by the ABTS and DPPH radical-scavenging assays and the SOD- and CAT-like activity measurements.

Taken together, these results demonstrate that AS@PB-CTP possesses broad radical-scavenging activity together with SOD-, CAT-, and POD-like catalytic functions. In particular, its ability to eliminate superoxide radicals and H_2_O_2_ provides a biochemical basis for alleviating oxidative stress, while the context-dependent POD-like behavior further reflects the catalytic versatility of the nanozyme. These properties provide a solid biochemical foundation for the subsequent evaluation of AS@PB-CTP in cellular and in vivo models of ICI-associated myocarditis.

### 2.4 Preferential cardiomyocyte uptake of FITC-labeled AS@PB-CTP

To visualize intracellular uptake, the fluorescent probe FITC was adsorbed onto AS@PB-CTP nanostructures to obtain FITC-labeled AS@PB-CTP. Following removal of unbound FITC, the fluorescently labeled formulation was used for cellular uptake studies. HL-1 cardiomyocytes were incubated with FITC-labeled AS@PB-CTP and imaged by confocal laser scanning microscopy (CLSM) at different time points (0, 2, 4, and 8 h). CLSM images showed almost no fluorescence at baseline, whereas a pronounced, time-dependent increase in intracellular green fluorescence was observed after exposure to FITC-labeled AS@PB-CTP, with punctate signals predominantly located in the perinuclear region (Fig. S4a). These results indicate efficient internalization and gradual accumulation of AS@PB-CTP within cardiomyocytes, providing a mechanistic basis for direct myocardial delivery and cytoprotective action in autoimmune myocarditis. To assess the cell-type preference conferred by CTP, the uptake of fluorescently labeled AS@PB and AS@PB-CTP was compared in HL-1 cardiomyocytes and L929 fibroblast-like cells. CTP modification had little effect on nanoparticle uptake in L929 cells but significantly enhanced uptake in HL-1 cardiomyocytes (Fig. S4b and c). This differential uptake pattern indicates that CTP functionalization preferentially promotes nanoparticle internalization by cardiomyocytes.

### 2.5 In vitro antioxidant and immunoregulatory effects

**Fig. 4:**
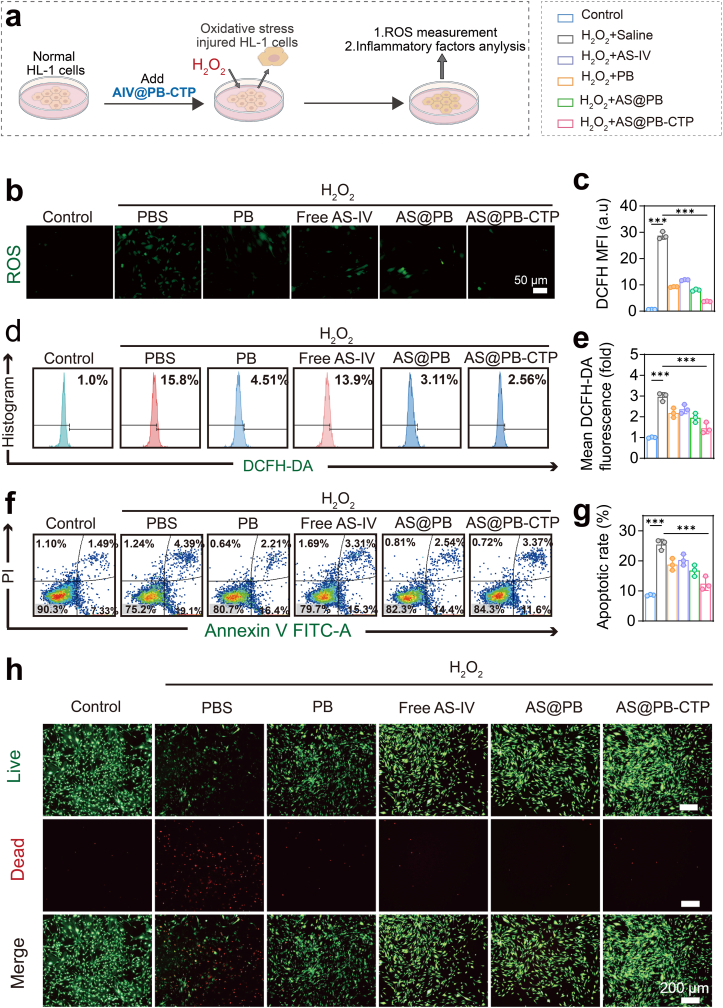
**AS@PB-CTP alleviates oxidative-stress–induced ROS overload and apoptosis in HL-1 cardiomyocytes.** (a) Experimental workflow for oxidative-stress and inflammatory readouts in H_2_O_2_-challenged HL-1 cardiomyocytes. (b,c) Representative DCFH-DA fluorescence micrographs (b) and flow-cytometric quantification of DCFH mean fluorescence intensity (MFI) (c) showing reduced intracellular ROS after AS@PB-CTP. (d,e) Flow-cytometry histograms (d) and corresponding mean DCFH-DA fluorescence (fold change) (e) further confirming ROS scavenging. (f, g) Annexin V–FITC/PI staining plots (f) and quantification of apoptotic rate (g) indicating attenuated H_2_O_2_-induced apoptosis with AS@PB-CTP treatment. (h) Calcein-AM/PI live–dead staining demonstrating improved cell viability under oxidative stress. Scale bars: 50 μm (b) and 200 μm (h). One-way ANOVA with Tukey's post hoc test was used, *P < 0.05, **P < 0.01, and ***P < 0.001; statistical comparisons are indicated by the connecting lines.

ICI-associated myocarditis is characterized by excessive myocardial oxidative stress, which may contribute to DNA damage, calcium dysregulation, mitochondrial dysfunction, and cardiomyocyte apoptosis, thereby promoting disease progression. Therefore, based on a previously reported H_2_O_2_-induced oxidative stress model in HL-1 cardiomyocytes [15], with appropriate modifications to the experimental conditions, we established complementary in vitro models, including an H_2_O_2_-induced oxidative stress model in HL-1 cardiomyocytes and an LPS-stimulated inflammatory model in macrophages. These models recapitulate selected oxidative and inflammatory components of the myocarditis microenvironment and provide controllable platforms for evaluating the antioxidant, cytoprotective, and immunoregulatory effects of AS@PB-CTP (Fig. 4a). Given that CTP promotes preferential myocardial enrichment rather than exclusive cardiomyocyte targeting, AS@PB-CTP may also directly interact with inflammatory macrophages within the diseased myocardium. Therefore, an LPS-stimulated macrophage activation model was included to assess its direct immunoregulatory effects on these cells [[27], [28], [29]].

In H_2_O_2_-challenged HL-1 cardiomyocytes, DCFH-DA staining and flow cytometry revealed a marked increase in intracellular ROS levels following H_2_O_2_ exposure, while treatment with AS@PB-CTP significantly attenuated ROS accumulation compared with the free AS-IV and PB treatment groups (Fig. 4b–e). To further assess its anti-apoptotic activity, we performed Annexin V–FITC/PI dual staining. AS@PB-CTP markedly decreased the percentages of both early and late apoptotic cardiomyocytes relative to the H_2_O_2_ group (Fig. 4f and g), indicating a strong capacity to mitigate H_2_O_2_-induced apoptosis. Live/dead staining further confirmed this cytoprotective effect, with AS@PB-CTP maintaining the highest proportion of viable cells among the tested treatment groups (Fig. 4h). Quantitative analysis of live and dead HL-1 cardiomyocytes further supported these observations (Fig. S5a and b). To further confirm the cytoprotective effect of AS@PB-CTP, a CCK-8 assay was performed in the HL-1 oxidative stress model. H_2_O_2_ exposure markedly reduced cell viability, confirming the successful induction of oxidative injury. In contrast, AS@PB-CTP treatment significantly restored HL-1 cell viability under H_2_O_2_-induced stress (Fig. S5c). Together, these results indicate that AS@PB-CTP not only reduces intracellular ROS accumulation but also limits apoptosis and preserves cardiomyocyte viability under oxidative stress conditions.

**Fig. 5:**
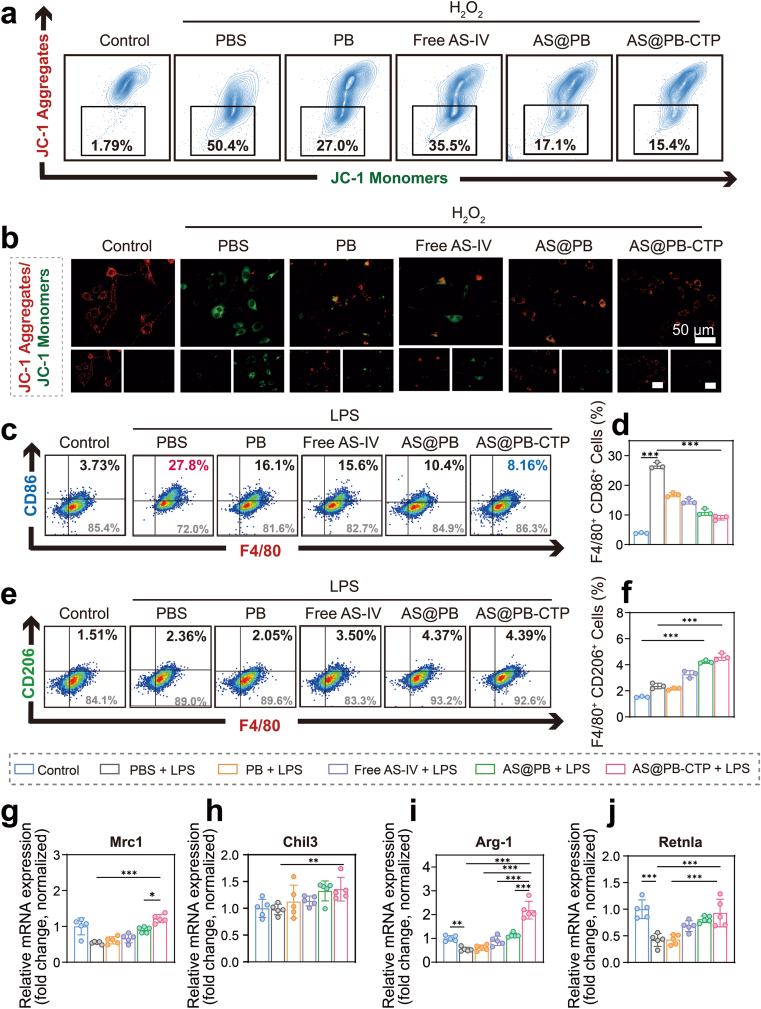
**In-vitro antioxidant cytoprotection and macrophage polarization shift mediated by AS@PB-CTP.** (a, b) JC-1 flow cytometry (a) and fluorescence imaging (b) demonstrating restoration of mitochondrial membrane potential, reflected by increased JC-1 aggregates and decreased monomers after AS@PB-CTP. (c–f) Flow-cytometric analysis of macrophage polarization (gated on F4/80^+^ cells): AS@PB-CTP decreases CD86^+^ (M1-like) and increases CD206^+^ (M2-like) subsets; bar graphs summarize frequencies. (g–j) qRT-PCR of M2-associated genes (*Mrc1, Chil3, Arg1, Retnla*) showing transcriptional upregulation consistent with anti-inflammatory, reparative polarization. Scale bars: 50 μm in (b). One-way ANOVA with Tukey's post hoc test was used, *P < 0.05, **P < 0.01, and ***P < 0.001; statistical comparisons are indicated by the connecting lines.

In parallel, JC-1 staining demonstrated that mitochondrial membrane potential was largely preserved after AS@PB-CTP treatment, as reflected by a reduced green/red fluorescence ratio compared with the H_2_O_2_ group, indicating attenuation of H_2_O_2_-induced mitochondrial membrane depolarization (Fig. 5a and b and Fig. S5d). In LPS-stimulated macrophages, AS@PB-CTP exerted notable immunomodulatory effects by modulating macrophage-associated inflammatory phenotype**s.** Flow cytometry analysis showed a pronounced reduction in the proportion of CD86^+^ M1-like macrophages after AS@PB-CTP treatment (Fig. 5c and d), accompanied by a concomitant increase in CD206^+^ M2-like macrophages (Fig. 5e and f). Consistently, quantitative real-time polymerase chain reaction (qRT-PCR) analysis revealed significant upregulation of M2-associated genes, including *Mrc1, Arg1, Chil3 (Ym1), and Retnla (Fizz1),* following AS@PB-CTP exposure (Fig. 5g–j), supporting a shift toward an M2-like phenotype. Together with the demonstrated cellular uptake of AS@PB-CTP by cardiomyocytes, these data indicate that AS@PB-CTP effectively scavenges intracellular ROS, preserves mitochondrial membrane potential, attenuates apoptosis, and modulates macrophage phenotypes toward an M2-like phenotype under the tested conditions. This dual action on cardiac parenchymal cells and immune cells provides a mechanistic basis for evaluating whether AS@PB-CTP can attenuate the mutually reinforcing relationship between oxidative injury and inflammation in vivo.

### 2.6 In vivo targeted delivery, structural protection, and functional restoration by AS@PB-CTP

**Fig. 6:**
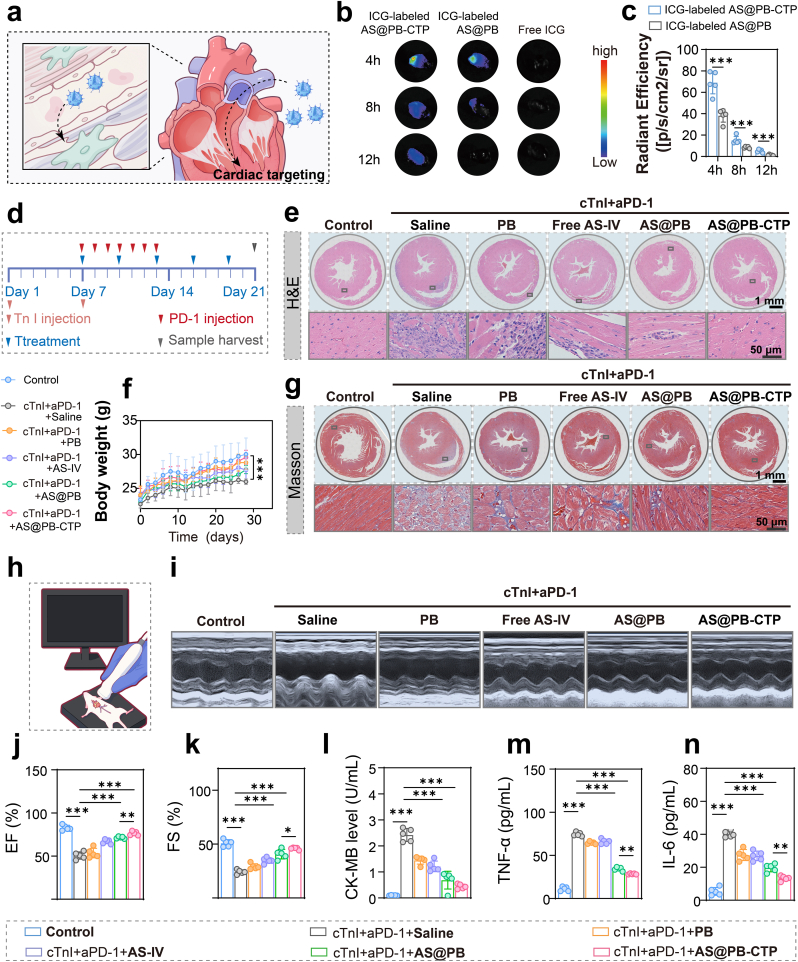
**In vivo biodistribution and therapeutic efficacy of AS@PB-CTP in the anti-PD-1/cTnI myocarditis model.** (a) Experimental schematic for biodistribution and therapy evaluation. (b) Ex vivo near-infrared fluorescence of hearts at 4, 8, and 12 h after i. v. Injection of ICG-labeled formulations (AS@PB-CTP, AS@PB) or free ICG. (c) Quantification of cardiac radiant efficiency showing superior targeting/retention of AS@PB-CTP. (d) Timeline of PD-1/TnI immunization, anti-PD-1 dosing, treatment, and sample collection. (e) Representative H&E sections showing reduced inflammatory infiltration with AS@PB-CTP versus controls. (f) Body-weight trajectories during treatment indicating good systemic tolerability. (g) Masson trichrome staining demonstrating attenuation of interstitial fibrosis by AS@PB-CTP. (h) Schematic of echocardiography acquisition. (i) Representative M-mode echocardiograms from each group. (j, k) Quantification of cardiac function: ejection fraction (EF), fractional shortening (FS). (l) Serum CK-MB and (m, n) circulating cytokines (TNF-α, IL-6) showing reduced myocardial injury and systemic inflammation after AS@PB-CTP. Scale bars: 1 mm (overview) and 50 μm (insets) in (e, g). One-way ANOVA with Tukey's post hoc test was used, *P < 0.05, **P < 0.01, and ***P < 0.001; statistical comparisons are indicated by the connecting lines.

Building on the in vitro cytoprotective and immunoregulatory effects of AS@PB-CTP, its in vivo biodistribution and therapeutic efficacy were subsequently evaluated (Fig. 6a). Because CTP was incorporated to promote preferential enrichment of the nanoformulation in injured myocardial tissue, near-infrared fluorescence imaging was first used to assess the cardiac distribution of ICG-labeled formulations. Ex vivo images revealed markedly stronger cardiac fluorescence in mice treated with ICG-labeled AS@PB-CTP than in those receiving ICG-labeled AS@PB or free ICG at 4, 8, and 12 h post-injection (Fig. 6b). Quantitative analysis confirmed significantly increased cardiac radiant efficiency in the AS@PB-CTP group, which peaked at 4h and then declined gradually while remaining above that of the other groups over the 12 h observation period (Fig. 6c). To further characterize the organ distribution of the formulations, free ICG, AS@PB-ICG, and AS@PB-CTP-ICG were intravenously administered, followed by ex vivo fluorescence imaging of the heart, liver, spleen, lungs, and kidneys at 0, 4, 8, 12, 24, and 48 h. The fluorescence signal was predominantly detected in the liver at the early time points, while detectable signals were also observed in the heart, spleen, lungs, and kidneys. Quantitative analysis at 4 and 24 h confirmed concurrent cardiac and splenic exposure, and the organ-associated signals gradually decreased during the observation period. These findings provide an anatomical context for the local cardiac effects and accompanying peripheral immune alterations (Fig. S6). These data indicate that CTP functionalization confers efficient myocardial homing and sustained retention. Based on the established TnI-induced experimental autoimmune myocarditis (TnI-AM) model that recapitulates heart-specific autoimmunity and shares key immunopathologic features with ICI-related myocarditis, TnI immunization was combined with PD-1 blockade in BALB/c mice to establish a pharmacological ICI-myocarditis-like model (Fig. 6d). Histopathological evaluation further substantiated the cardioprotective effects of AS@PB-CTP. H&E staining showed extensive inflammatory cell infiltration and cardiomyocyte disarray in saline-treated myocarditis hearts, whereas these lesions were attenuated to varying degrees in PB-, AS-IV-, and AS@PB-treated groups and most markedly attenuated after AS@PB-CTP administration (Fig. 6e). Induction of ICI myocarditis markedly blunted weight gain in saline-treated mice, whereas PB, Free AS-IV, AS@PB and AS@PB-CTP all partially preserved body weight over the 21-day period. Notably, AS@PB-CTP–treated animals followed a body-weight trajectory comparable to the other treatment groups and clearly above the saline group, with no evident treatment-related body-weight loss(Fig. 6f). Masson's trichrome staining demonstrated pronounced interstitial fibrosis and collagen deposition in the saline group, which were substantially reduced in the AS@PB-CTP group, with preservation of myocardial architecture (Fig. 6g–S7), indicating attenuation of adverse structural remodeling. We used transthoracic echocardiography to assess heart function (Fig. 6h and i). Mice with ICI-induced myocarditis that received only saline showed clear signs of damage. Their left ventricular ejection fraction (EF) and fractional shortening (FS) were markedly reduced, indicating impaired systolic function. AS@PB-CTP treatment produced the greatest improvement in EF and FS among the tested formulations (Fig. 6j and k). Serum biochemical analysis provided additional evidence of myocardial protection. CK-MB levels were markedly elevated in saline-treated model mice but were significantly reduced following AS@PB-CTP treatment (Fig. 6l). Serum TNF-α and IL-6 levels were also markedly decreased in the AS@PB-CTP group (Fig. 6m and n), indicating reduced systemic inflammatory activity together with alleviation of myocardial injury. Overall, these results show that AS@PB-CTP accumulates in the injured heart and provides strong protection. It improves heart structure and function by reducing inflammation and fibrosis. These features make it a promising nanoplatform for treating immune-related heart injury.

### 2.7 Transcriptomic analysis of molecular mechanisms

**Fig. 7:**
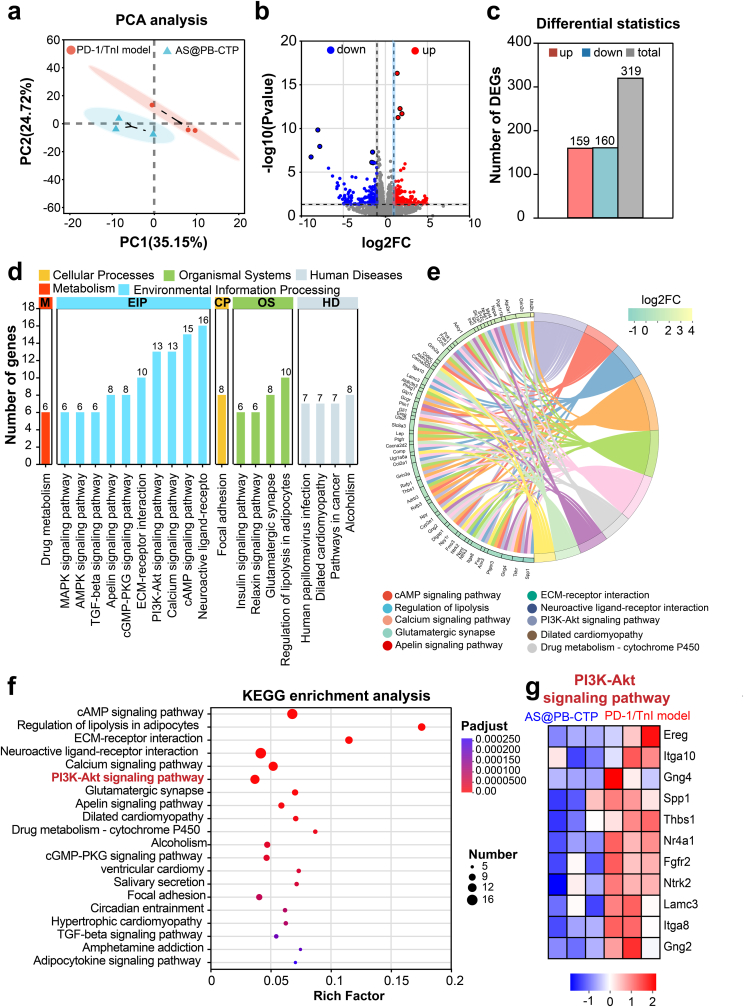
**Cardiac transcriptomics in the anti-PD-1/cTnI myocarditis model reveal PI3K–Akt pathway modulation by AS@PB-CTP.** (a) PCA of heart RNA-seq profiles showing clear separation between the anti-PD-1/cTnI myocarditis model and AS@PB-CTP–treated mice. (b,c) Differential expression analysis between the two groups, displayed as a volcano plot (b) and bar summary (c), identifying 319 DEGs (159 up- and 160 down-regulated; significance cutoffs as indicated in the plot). (d) Functional categorisation of enriched pathways across Metabolism, Cellular Processes, Organismal Systems, Environmental Information Processing, and Human Diseases. (e) Chord diagram linking key DEGs to enriched pathways, underscoring the central role of PI3K–Akt signalling in AS@PB-CTP–mediated cardioprotection. (f) KEGG pathway enrichment of DEGs highlighting pathways linked to oxidative stress and immune regulation, with PI3K–Akt signalling among the top hits. (g) Heat map of representative PI3K–Akt–related genes (e.g., *Ereg, Itga10, Gng4, Spp1, Thbs1, Nr4a1, Fgfr2, Ntrk2, Lamc3, Gng2, Itga8*).

To better understand how AS@PB-CTP works at the molecular level, we performed RNA sequencing on heart tissues from treated and untreated mice. Principal component analysis (PCA) showed clear differences in gene expression patterns between the two groups (Fig. 7a). Differential expression analysis identified 319 differentially expressed genes (DEGs) after treatment, including 159 upregulated genes and 160 downregulated genes (Fig. 7b and c). These findings demonstrate substantial treatment-associated transcriptional remodeling [17,30]. Among the top enriched signaling pathways, cAMP, calcium, ECM–receptor interaction and PI3K–Akt signaling were prominently represented, together with neuroactive ligand–receptor interaction, apelin signaling and cardiomyopathy-associated pathways (Fig. 7d). The chord diagram further illustrated the distribution and overlap of DEGs among these major enriched pathways, with PI3K–Akt signaling emerging as one of the prominently represented pathways (Fig. 7e). KEGG functional classification additionally indicated that the affected genes were related to cellular communication, tissue remodeling, metabolism, and disease-related processes (Fig. 7f). Focusing on the PI3K–Akt pathway, heatmap visualization highlighted a set of hub pathway-associated genes—including *Ereg, Itga10, Gng4, Spp1, Thbs1, Nr4a1, Fgfr2, Ntrk2, Lamc3, Itga8 and Gng2*—that were markedly dysregulated in myocarditis hearts and shifted toward control-like expression patterns following AS@PB-CTP treatment (Fig. 7g). Collectively, these transcriptomic findings indicate that AS@PB-CTP treatment is associated with coordinated regulation of inflammatory, extracellular-matrix, repair-related, and metabolic transcriptional programs, with PI3K–Akt signaling representing a prominent treatment-associated molecular node.

**Fig. 8:**
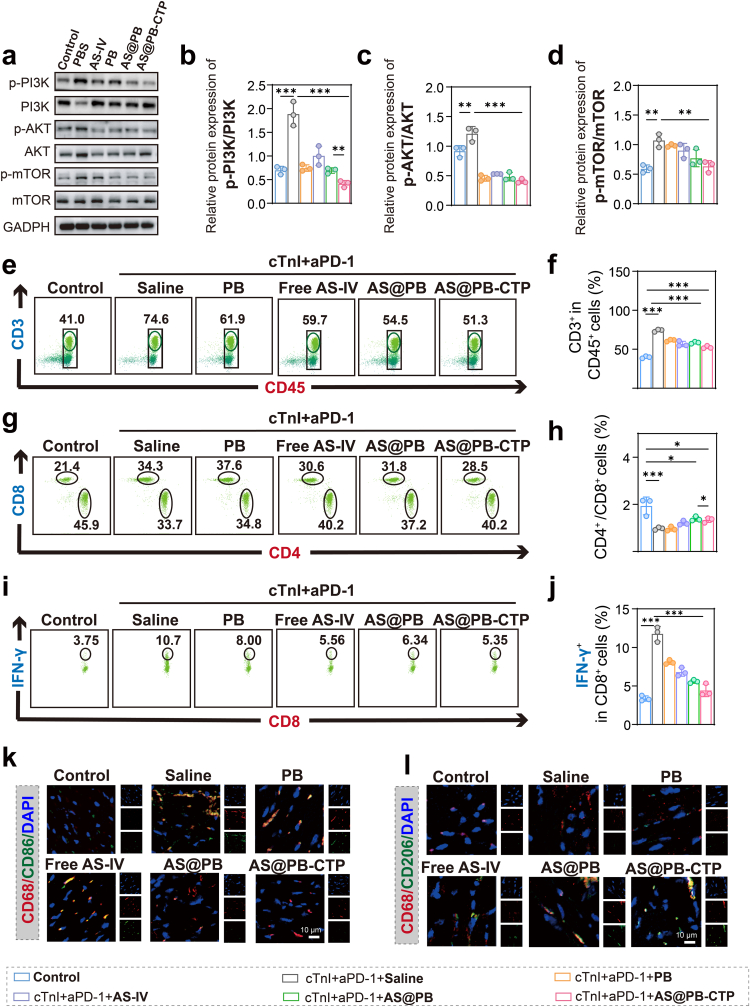
**AS@PB-CTP suppresses cardiac PI3K–Akt–mTOR activation and mitigates systemic proinflammatory T-cell/macrophage programs in the anti-PD-1/cTnI myocarditis model of ICI-myocarditis.** (a)Representative immunoblots of PI3K–Akt–mTOR signaling (p-PI3K/PI3K, p-AKT/AKT, p-mTOR/mTOR); GAPDH serves as the loading control. (b–d) Densitometric quantification (phospho/total ratios, normalized to GAPDH) showing significant reductions of p-PI3K/PI3K (b), p-AKT/AKT (c), and p-mTOR/mTOR (d) after AS@PB-CTP treatment, indicating pathway deactivation. (e,f) Representative flow-cytometry plots of splenic CD3^+^ T cells within CD45^+^ leukocytes (e) and corresponding quantification (f). (g, h) Representative plots of CD4^+^ and CD8^+^ T-cell subsets within splenic T cells (g) and quantification of the CD4^+^/CD8^+^ ratio (h). (i,j) Representative plots of IFN-γ^+^ cells within the CD8^+^ T-cell compartment (i) and summary quantification (j), indicating contraction of proinflammatory IFN-γ–producing CD8^+^ effector cells after AS@PB-CTP. (k, l) Cardiac immunofluorescence co-staining showing macrophage phenotypes: (k) CD68^+^CD86^+^ M1-like cells and (l) CD68^+^CD206^+^ M2-like cells (DAPI, nuclei). AS@PB-CTP decreases M1-like and increases M2-like macrophages in myocardium. Scale bars, 10 μm. One-way ANOVA with Tukey's post hoc test was used, *P < 0.05, **P < 0.01, and ***P < 0.001; statistical comparisons are indicated by the connecting lines.

To validate these findings at the gene and protein levels, representative PI3K–Akt-associated genes were first examined by quantitative real-time PCR. qPCR analysis confirmed that the inflammation- and remodeling-associated genes *Gng4, Spp1, Thbs1,* and ***Nr4a1*** were robustly upregulated in PBS-treated myocarditis hearts and significantly downregulated after AS@PB-CTP treatment (Fig. S8a–d). Similarly, the expression patterns of *Ereg, Itga10, Fgfr2, Ntrk2, Lamc3, Itga8,* and *Gng2* shifted toward those observed in healthy controls after treatment (Fig. S8e–k). These results were consistent with the RNA-sequencing data and further supported broad treatment-associated modulation of the myocardial inflammatory and remodeling microenvironment. Consistent with the transcriptional changes, Western blot analysis showed pronounced activation of the PI3K–Akt–mTOR axis in myocarditis hearts, as reflected by increased phosphorylation of PI3K, AKT and mTOR in the PBS group. AS@PB-CTP treatment markedly reduced the levels of p-PI3K, p-AKT and p-mTOR, while total PI3K, AKT and mTOR remained relatively unchanged (Fig. 8a). Densitometric quantification of p-PI3K/PI3K, p-AKT/AKT and p-mTOR/mTOR ratios confirmed significant reductions in pathway phosphorylation following AS@PB-CTP treatment (Fig. 8b–d). Among the tested formulations, AS@PB-CTP produced the most pronounced reduction in pathway phosphorylation, consistent with its enhanced therapeutic effects observed in vivo.

When interpreted together with the preceding network pharmacology and molecular docking results, these experimental findings provide multi-level evidence linking the PI3K–Akt–mTOR axis to the AS@PB-CTP treatment response. Taken together, these findings support the PI3K–Akt–mTOR axis as a mechanistically relevant signaling node associated with AS@PB-CTP treatment, with its suppression occurring in parallel with reduced oxidative stress, attenuated inflammatory activation, improved cardiomyocyte survival, and alleviated fibrotic remodeling.

### 2.8 Coordinated immune modulation in the spleen and myocardium

ICI-associated myocarditis is characterized by dysregulated T-cell responses and the accumulation of proinflammatory myeloid cells within the heart. Inflammatory monocytes are generated primarily in the bone marrow, while the spleen can serve as an extramedullary reservoir from which monocytes are mobilized into the circulation in response to inflammatory signals [[31], [32], [33]]. Once recruited to the injured heart, circulating monocytes can differentiate into macrophages and contribute to the myocardial macrophage compartment [34]. To determine whether AS@PB-CTP treatment was associated with coordinated immune changes in peripheral lymphoid tissue and the myocardium, splenic T-cell populations and cardiac macrophage phenotypes were subsequently examined. Flow cytometric profiling of splenic leukocytes showed a marked expansion of CD3^+^ T cells within the CD45^+^ compartment in PBS-treated model mice, while PB or Free AS-IV monotherapy produced only modest reductions (Fig. 8e and f). In contrast, AS@PB-CTP substantially reduced the CD3^+^ fraction toward control levels, indicating attenuation of the increased splenic T-cell abundance observed in the model group. Subset analysis demonstrated that myocarditis shifted the splenic T-cell pool toward a higher proportion of CD8^+^ T cells and a lower proportion of CD4^+^ T cells, resulting in a decreased CD4^+^/CD8^+^ ratio (Fig. 8g and h). Treatment with AS@PB-CTP largely restored this balance by lowering the proportion of CD8^+^ cells and increasing CD4^+^ cells within the CD3^+^ population (Fig. S9a and b). These changes indicate partial normalization of the splenic CD4^+^/CD8^+^ T-cell distribution rather than generalized depletion of the T-cell compartment. To further assess inflammatory effector activity, we performed intracellular cytokine staining for IFN-γ within the CD8^+^ T-cell compartment. Saline-treated the anti-PD-1/cTnI myocarditis model mice showed a pronounced increase in IFN-γ^+^ CD8^+^ T cells compared with healthy controls, consistent with an amplified Tc1-like response and a systemic bias toward cytotoxic inflammation (Fig. 8i and j). AS@PB-CTP treatment significantly reduced the proportion of IFN-γ-producing CD8^+^ T cells. This reduction was greater than that observed with PB, free AS-IV, or AS@PB under the tested conditions. Together with the changes in CD4^+^ and CD8^+^ T-cell distribution, these findings support attenuation of the splenic proinflammatory CD8^+^ effector response following AS@PB-CTP treatment.

The effects of AS@PB-CTP on cardiac macrophage-associated phenotypes were next evaluated. Cardiac sections from PBS-treated model mice showed abundant CD68^+^CD86^+^ M1-like macrophages and relatively fewer CD68^+^CD206^+^ M2-like cells (Fig. 8k and l). AS@PB-CTP treatment reduced the density of CD68^+^CD86^+^ cells and increased the relative abundance of CD68^+^CD206^+^ cells, supporting a shift from a predominantly proinflammatory macrophage-associated phenotype toward an M2-like reparative phenotype. These changes were more pronounced than those observed in the PB, free AS-IV, and AS@PB groups when supported by the corresponding quantitative comparisons. To further characterize macrophage populations associated with tissue residency and inflammatory recruitment, cardiac sections were additionally stained for CD68, TIM4, and CCR2. TIM4 is commonly associated with subsets of resident cardiac macrophages, whereas CCR2 is enriched in inflammatory monocytes and recruited monocyte-derived macrophages. Representative immunofluorescence images showed strong CD68 and CCR2 signals with relatively weak TIM4 staining in the saline-treated myocarditis group, consistent with increased accumulation of recruited inflammatory macrophage-like cells (Fig. S10). In contrast, AS@PB-CTP treatment markedly reduced the CD68^+^CCR2^+^ signal and was associated with relative preservation or enrichment of CD68^+^TIM4^+^ cells, suggesting a shift in the cardiac macrophage compartment from a recruited inflammatory phenotype toward a resident-like, tissue-supportive phenotype. Collectively, AS@PB-CTP treatment was associated with concurrent attenuation of splenic proinflammatory T-cell responses and cardiac macrophage-associated inflammation. These coordinated immune alterations may contribute to the alleviation of the sustained inflammatory microenvironment underlying immune-mediated myocardial injury, although the present data do not establish a causal relationship between splenic immune changes and myocardial immune remodeling.

### 2.9 Biocompatibility and systemic safety evaluation of AS@PB-CTP

To assess in vitro biocompatibility, AS@PB-CTP exhibited negligible cytotoxicity in HL-1 cardiomyocytes and RAW264.7 macrophages across a wide dose range (0–200 μg/mL), maintaining cell viability above 90% (Fig. S11a and b). H&E staining of major organs (heart, liver, spleen, lung, and kidney) showed no histopathological abnormalities following administration, indicating favorable in vivo compatibility (Fig. S12). In addition, serum biochemistry demonstrated no significant alterations in renal function markers (CREA), blood urea nitrogen (BUN) and hepatic function markers (AST, ALT). Routine hematological analysis also showed comparable red blood cell, white blood cell, hemoglobin, and platelet levels between AS@PB-CTP-treated mice and control mice (Figs. S13–S14). Collectively, these findings indicate that systemic administration of AS@PB-CTP did not cause detectable acute hepatic, renal, hematological, or histopathological abnormalities under the evaluated conditions. To evaluate the long-term in vivo fate of AS@PB-CTP, Mn, an intrinsic element within the PB-based nanozyme framework, was used as a tracer for material-associated elemental signals in cardiac tissue and blood. Mn levels were quantified at 0 h, 24 h, Day 7, and Day 28 after administration. A marked increase in Mn levels was observed at 24 h, followed by a gradual decline toward baseline by Day 28 (Fig. S15a and b). These results indicate a transient in vivo presence of AS@PB-CTP-associated elemental signals, with no evident long-term Mn accumulation in blood or cardiac tissue. At Day 28, no significant alterations were detected in serum biochemical or hematological parameters, and H&E staining of the heart, liver, spleen, lungs, and kidneys revealed no obvious pathological abnormalities (Fig. S15c–l). Together, these findings suggest that AS@PB-CTP achieved effective early cardiac exposure while avoiding prolonged Mn-associated retention in blood or cardiac tissue, with no detectable delayed systemic toxicity during the 28-day observation period.

## 3 Discussion

The present findings position AS@PB-CTP as a cardiac-targeted nanozyme platform that integrates catalytic redox regulation with immune modulation to attenuate immune-mediated myocardial injury. In vitro, it curtailed ROS accumulation in HL-1 cardiomyocytes, preserved mitochondrial membrane potential, and reduced apoptosis, while also modulating macrophages from a CD68^+^CD86^+^ M1-like phenotype toward a CD68^+^CD206^+^ M2-like state. In the anti-PD-1/cTnI myocarditis model, systemically administered AS@PB-CTP preferentially accumulated in the inflamed myocardium, attenuated leukocyte infiltration and interstitial fibrosis, and restored ventricular function. Transcriptomic and protein-level analyses further identified the PI3K–Akt–mTOR axis as a treatment-associated signaling node, accompanied by downregulation of inflammatory and remodeling-related mediators. These findings suggest that the therapeutic effects of AS@PB-CTP involve coordinated redox protection and immune microenvironment modulation, rather than simple ROS scavenging alone. Compared with non-targeted AS@PB, CTP conjugation conferred a key targeting advantage by markedly enhancing myocardial accumulation of the nanozyme platform in the inflamed heart. This heart-directed enrichment is critical because it increases local therapeutic exposure at the site of immune-mediated injury, thereby amplifying the cardioprotective effects of AS@PB in terms of ventricular functional recovery and inflammatory cytokine suppression. Therefore, the main benefit of CTP modification is not expected to appear as uniform superiority across every downstream molecular or immune marker, but rather as improved myocardial delivery that translates into stronger overall therapeutic performance.

These data should be interpreted in the context of accumulating evidence that has reframed ICI myocarditis as an antigen-driven autoimmune process rather than a nonspecific drug toxicity. In troponin I–induced experimental autoimmune myocarditis, cardiac TnI immunization elicits myocarditis with T-cell activation, cardiac autoimmunity, and inflammatory transcriptional programs that overlap with key features of human ICI-associated myocarditis [35]. Building on this concept, recent studies have extended the rationale for immunometabolic intervention in ICI-associated myocarditis. For example, crocin and L-kynurenine, an IDO1-derived metabolite, have been reported to exert protective effects in cTnI/PD-1-based mouse models. These effects were associated with reduced pathogenic T-cell and macrophage responses, supporting the feasibility of targeting downstream immune-inflammatory injury in this disease setting. Accordingly, a related TnI/PD-1 protocol was used in BALB/c mice in the present study to establish a reproducible ICI-myocarditis-like model. Although this model does not fully recapitulate the clinical complexity of ICI-associated myocarditis, it captures key features of cardiac inflammation and immune dysregulation and therefore provides a useful experimental platform for evaluating nanozyme-based redox–immune intervention [36].

The PI3K–Akt–mTOR pathway is a central signaling network that integrates inflammatory activation, metabolic stress, cell survival, and tissue remodeling in cardiovascular disease. Its biological output is highly dependent on cellular context and activation status: appropriately regulated Akt signaling can support cardiomyocyte survival under stress, whereas sustained PI3K–Akt–mTOR activation in the inflamed myocardial microenvironment may amplify immune activation, stromal remodeling, and pathological tissue responses [37,38]. Recent evidence further supports the relevance of this pathway in ICI-associated myocarditis. Single-cell and spatial profiling of human ICI-associated myocarditis has revealed enrichment of cytotoxic T cells, T/NK-cell activation, mononuclear phagocytes, inflammatory fibroblasts, IFN-γ-related responses, antigen-presentation programs, and CXCL9/CXCL10 expression in affected myocardium [4]. Notably, mTORC1-related transcriptional activity in intracardiac T/NK cells was associated with serum troponin T levels, suggesting a potential relationship between mTOR-related immune activation and the severity of myocardial injury in ICI-associated myocarditis. In addition, mechanistic studies indicate that ICI-mediated cardiac injury involves autoreactive T-cell activation and subsequent amplification through innate immune responses, forming a pathogenic T-cell–myeloid inflammatory circuit [39]. Beyond T-cell responses, dysregulated mTORC1 signaling in cardiac myeloid compartments has also been linked to macrophage accumulation, inflammatory infiltration, interstitial and perivascular fibrosis, gap-junction remodeling, and cardiac dysfunction, supporting a potential role of myeloid PI3K–Akt–mTOR activity in macrophage-associated inflammation and remodeling [40,41]. These findings provide a clinically and mechanistically relevant framework for interpreting PI3K–Akt–mTOR-related changes in the present study.

In this study, RNA-seq analysis identified PI3K–Akt-related transcriptional changes as one of the prominent treatment-associated pathways, and Western blotting further showed increased phosphorylation of PI3K, AKT, and mTOR in myocarditis hearts. AS@PB-CTP treatment markedly reduced these phosphorylation signals, accompanied by decreased inflammatory activation, improved cardiomyocyte survival, and alleviated fibrotic remodeling. These coordinated changes suggest that AS@PB-CTP helps shift the injured myocardium away from a sustained proinflammatory and pro-remodeling signaling state. Together with network pharmacology and molecular docking analyses indicating potential interactions between AS-IV and pathway-associated targets, these findings support PI3K–Akt–mTOR signaling as a mechanistically relevant node linking the redox-regulatory, immunomodulatory, and tissue-protective effects of AS@PB-CTP. Nevertheless, because the current evidence is mainly based on transcriptomic enrichment, protein-level validation, network pharmacology, and molecular docking, these results should be interpreted as correlative and predictive rather than definitive causal evidence. Future studies using pathway-specific inhibition, activation, genetic intervention, or rescue experiments will be required to determine whether PI3K–Akt–mTOR signaling directly mediates the therapeutic efficacy of AS@PB-CTP.

The present findings further suggest that AS@PB-CTP treatment is associated with coordinated immune modulation between peripheral lymphoid compartments and the local myocardial microenvironment. The spleen is increasingly recognized not only as a reservoir of circulating immune cells, including T cells and inflammatory monocytes, but also as an active immune amplifier in cardiovascular injury [[42], [43], [44], [45]]. For example, in pressure-overload models, lactotransferrin released from stressed hearts has been reported to reprogram splenic B cells through the Ncl–ERK1/2–ADAM10–CD23 pathway, thereby promoting IgE overproduction and aggravating maladaptive cardiac hypertrophy and fibrosis [43]. Although this B cell–IgE-centered mechanism differs from the T cell- and macrophage-dominant inflammatory features of ICI-associated myocarditis, it highlights the broader principle that injured myocardium can communicate with the spleen and shape peripheral immune outputs that subsequently influence cardiac remodeling. In this study, AS@PB-CTP reduced the splenic CD3^+^ T-cell fraction, partially normalized the CD4^+^/CD8^+^ T-cell distribution, and attenuated the IFN-γ^+^CD8^+^ effector response. In parallel, AS@PB-CTP reduced CD68^+^CD86^+^ M1-like macrophage signals, increased CD68^+^CD206^+^ M2-like signals, reduced CD68^+^CCR2^+^ inflammatory macrophage-like signals, and relatively preserved CD68^+^TIM4^+^ resident-like macrophage populations in the myocardium. These synchronized changes suggest that AS@PB-CTP may coordinately attenuate peripheral proinflammatory T-cell activity and cardiac macrophage-associated inflammation. Together with growing evidence for heart–spleen immune communication in inflammatory cardiac remodeling, these findings support cardiosplenic immune crosstalk as a plausible component of the AS@PB-CTP treatment response. These observations also open a future direction for exploring how cardiac-targeted redox–immune nanomedicine may coordinate peripheral immune regulation with local myocardial protection through peripheral–cardiac immune communication. However, these findings should be interpreted as coordinated splenic and cardiac immune changes rather than definitive evidence of a causal cardiosplenic axis, which will require further validation using immune-cell trafficking, splenectomy, adoptive transfer, or organ-specific intervention approaches.

Another important translational consideration is whether AS@PB-CTP-mediated immune modulation could influence the antitumor efficacy of ICIs. Clinically, ICIs are generally withheld once ICI-associated myocarditis is suspected or diagnosed, and high-dose corticosteroids remain the first-line treatment for clinically significant cases [46,47]. Therefore, in established ICI-associated myocarditis, the immediate therapeutic priority is to control potentially life-threatening cardiac immune injury. In this context, AS@PB-CTP is designed as a cardiac-targeted redox–immune nanoplatform rather than a broad systemic immunosuppressant, with the aim of attenuating pathological myocardial inflammation and oxidative injury while potentially reducing the need for prolonged or excessive systemic immunosuppression. Nevertheless, CD8^+^ effector T cells, IFN-γ-related signaling, and macrophage functional states also contribute to antitumor immunity during PD-1 blockade; thus, the possibility that broad or prolonged immunomodulation by AS@PB-CTP may affect ICI-mediated tumor control cannot be excluded. Recent work by Warrick et al. showed that ICI-induced myocarditis can be driven by autoreactive CD8^+^ T cell-derived TNF and TNFR2 signaling, and that genetic ablation of CD8^+^ T cell-derived TNF or TNFR2 blockade prevented cardiotoxicity while preserving antitumor efficacy [39]. This finding suggests that cardiotoxic inflammatory circuits may be at least partially separable from antitumor immune responses. However, AS@PB-CTP is not a TNFR2-specific inhibitor, and the present study was performed in a non-tumor-bearing PD-1/TnI model. Therefore, future studies in immunocompetent syngeneic tumor-bearing models are required to determine whether AS@PB-CTP can protect the heart without compromising ICI-mediated antitumor efficacy. This translational uncertainty also applies to the targeting component of the platform. Although CTP has shown cardiac-targeting potential in previous preclinical studies, the present work did not directly validate AS@PB-CTP uptake in human cardiomyocytes or human cardiac tissues. Future studies using human induced pluripotent stem cell-derived cardiomyocytes, human cardiac organoids, ex vivo human myocardium, and clinically relevant large-animal models will be required to determine whether CTP-mediated myocardial targeting can be translated to human applications.

Taken together, this study demonstrates that AS@PB-CTP provides a multifunctional nanozyme-based strategy for immune-mediated myocardial injury by integrating preferential cardiac delivery, catalytic ROS scavenging, mitochondrial protection, and immune microenvironment modulation. By attenuating cardiomyocyte oxidative stress, reducing splenic proinflammatory T-cell responses, and shifting cardiac macrophage-associated phenotypes toward a less inflammatory state, AS@PB-CTP interrupts multiple pathological processes involved in ICI-myocarditis-like injury. These findings support the potential of cardiac-targeted redox–immune nanomedicine as a therapeutic strategy for ICI-associated myocarditis and other immune-mediated inflammatory heart diseases.

## 4 Conclusion

In summary, we developed AS@PB-CTP as a cardiac-targeted nanozyme platform for coordinated redox regulation and immune modulation in immune-mediated myocardial injury. AS@PB-CTP effectively reduced intracellular ROS accumulation, preserved mitochondrial function, and attenuated cardiomyocyte apoptosis under oxidative stress. In parallel, the platform modulated macrophage-associated phenotypes toward a less inflammatory and more reparative-like state. In the anti-PD-1/cTnI myocarditis model, AS@PB-CTP preferentially accumulated in inflamed myocardial tissue, alleviated myocardial inflammation and fibrosis, and improved cardiac systolic function. Transcriptomic and protein-level analyses further identified the PI3K–Akt–mTOR axis as a treatment-associated signaling node, while immune profiling revealed coordinated attenuation of splenic proinflammatory T-cell responses and cardiac macrophage-associated inflammation. AS@PB-CTP also showed favorable biocompatibility and no detectable delayed systemic toxicity during the observation period. Collectively, these findings support AS@PB-CTP as a promising cardiac-targeted redox–immune nanotherapeutic strategy for ICI-associated myocarditis and other immune-mediated inflammatory heart diseases.

## 5 Materials and methods

### 5.1 Materials and reagents

Astragaloside IV (AS-IV, purity >98%), Nhydroxysulfosuccinimide (NHS) and 1-Ethyl-3-(3-dimethyl aminopropyl) carbodiimide hydrochloride (EDC) were obtained from the Aladdin Reagent Co. (Shanghai, China). K_4_[Fe(CN)_6_], bovine serum albumin (BSA), MnCl_2_, 1,2-distearoyl-sn-glycero-3-phosphoethanolamine conjugated polyethylene glycol acid (DSPE-PEG-COOH) and FITC were purchased from Sigma-Aldrich Trading Co., Ltd. (Shanghai, China). CTP (APWHLSSQYSRT) was supplied by GUOPING (Anhui China). All reagents and chemicals were of analytical grade and used as received without further purification. Calcein-AM/PI cell double staining kit was purchased from Dojindo (Kumamoto, Japan). BCA protein assay kit was purchased from EpiZyme Co., Ltd (Shanghai, China). Hydrogen peroxide (H_2_O_2_), 2,2′-azino-bis(3-ethylbenzothiazoline-6-sulfonic acid) (ABTS), and 1,1-diphenyl-2-picrylhydrazyl (DPPH) assay kits were obtained from Beyotime (Shanghai, China). Antibodies against AKT, p-AKT, mTOR, p-mTOR, p-PI3K, and PI3K were obtained from Cell Signaling Technology (USA).

### 5.2 Preparation and characterization of AS@PB–CTP

PB cores. BSA-stabilized Prussian blue nanoparticles were obtained by co-precipitating K_4_[Fe(CN)_6_]·3H_2_O (10 mL, 0.04 mmol) with MnCl_2_·6H_2_O (10 mL, 0.05 mmol) in an aqueous bovine serum albumin (BSA) solution under vigorous stirring at 60 °C for 2 h, followed by 12 h aging at room temperature. The nanoparticles were collected by centrifugation (≈14 000 × g, 10 min), washed with water three times, lyophilized to constant weight, and stored desiccated at 4 °C.

AS loading (AS@PB). Lyophilized PB was redispersed in PBS (pH 7.4) to 1.0 mg/mL (dry mass). AS-IV (2 mg) in PBS containing 1–2% (v/v) ethanol (10 mL) was added and the mixture shaken at 37 °C for 2–4 h, then equilibrated overnight. AS@PB was collected (≈14 000 ×g, 10 min) and washed with PBS (×3). AS-IV loading and EE% were quantified by HPLC (methods as for release studies).

PEGylation (AS@PB-PEG-COOH). DSPE–PEG(2000)–COOH (5 mg, DMSO stock) was added dropwise to AS@PB (10 mL, 1 mg/mL) and incubated at 60 °C for 2–4 h with gentle sonication (final DMSO ≤10% v/v). Particles were collected and redispersed in MES buffer (5 mL, 50 mM, pH 5.8–6.0).

CTP conjugation (AS@PB-CTP). Surface –COOH groups were activated with EDC (35 mg) and NHS (53 mg) in MES (pH 5.8–6.0) for 30–60 min (dark), followed by buffer exchange to PBS (pH 7.4). CTP (APWHLSSQYSRT; 4 mL, 2 mg/mL) was added and stirred overnight at room temperature. Residual NHS esters were quenched with ethanolamine (1 M, pH 8.0, 10–15 min). The product was collected, washed with water and ethanol, and redispersed in PBS (pH 7.4) for use.

We also made FITC-labeled AS@PB-CTP nanoparticles by simple adsorption. First, we prepared 10 mL of AS@PB-CTP dispersion (1 mg/mL). We then added 1 mL of FITC solution in DMSO to this dispersion. The mixture was stirred overnight at room temperature in the dark. After that, we collected the FITC-labeled nanoparticles by centrifugation. We washed the particles with deionized water and ethanol. Finally, we re-dispersed the particles in PBS.

### 5.3 Characterization

Transmission electron microscopy (TEM, JEOL JEM-2100) and scanning electron microscopy (SEM, Hitachi SU8020) were used to observe the morphology of AS@PB-CTP nanoparticles. Dynamic light scattering (DLS, Malvern Zetasizer Nano ZS90) was used to measure the hydrodynamic diameter and zeta potential. Fourier-transform infrared spectroscopy (FT-IR, Thermo Nicolet 6700) and X-ray photoelectron spectroscopy (XPS, Kratos AXIS Ultra DLD) were used to confirm surface chemical groups and Fe–N coordination. Ultraviolet–visible spectroscopy (UV–vis, Shimadzu UV-2600) was used to monitor the absorbance changes in antioxidant assays, including ABTS^•⁺^ and DPPH radical-scavenging experiments. High-performance liquid chromatography (HPLC) was used to quantify AS-IV in the nanoparticles and to calculate encapsulation efficiency (EE%). AS-IV release from AS@PB-CTP was tested in a constant-temperature shaker bath, and the released AS-IV was quantified by HPLC.

The CTP content in AS@PB-CTP was quantified using an indirect subtraction method. Briefly, after EDC/NHS-mediated coupling, the post-reaction supernatant and washing fractions were collected and analyzed using a BCA-based CTP standard curve to determine the amount of unreacted CTP. The amount of CTP associated with AS@PB-CTP was calculated by subtracting the unreacted CTP from the initial CTP input and was normalized to the final lyophilized mass of AS@PB-CTP nanoparticles. The CTP content was expressed as μg CTP per mg AS@PB-CTP and as wt% relative to the final nanoparticle mass.

### 5.4 ABTS^+•^ and DPPH free radical scavenging assays

The ABTS^+^• solution was prepared by mixing 7.4 mM ABTS with 2.6 mM K_2_S_2_O_8_ in PBS (pH 7.4) at a 1:1 vol ratio. The mixture was kept in the dark at 4 °C overnight and subsequently diluted with PBS to obtain an appropriate absorbance at 734 nm. AS@PB-CTP solutions were prepared at concentrations of 25, 50, 100, and 200 μg/mL. A 20 μL aliquot of each sample was mixed with 80 μL PBS and then added to 400 μL of the ABTS^+^• working solution. After incubation at room temperature for 5 min, the absorbance at 734 nm was measured using a UV–Vis spectrophotometer. Absorption spectra from 500 to 900 nm were also recorded. The ABTS^+^• scavenging efficiency was calculated from the decrease in absorbance relative to the blank control.

The radical-scavenging activity of AS@PB-CTP was further evaluated using the DPPH assay. AS@PB-CTP was dispersed in buffers at pH 6.5 or 7.4 and mixed with freshly prepared DPPH solution to obtain final concentrations of 0, 25, 50, 100, and 200 μg/mL. After incubation in the dark at room temperature for 30 min, UV–Vis absorption spectra were recorded from 400 to 700 nm, and the absorbance at 517 nm was used to calculate the DPPH radical-scavenging efficiency. All measurements were performed in triplicate.

### 5.5 Multienzyme-mimetic activity evaluation

The SOD-like activity of the nanozyme was evaluated using a commercial SOD assay kit based on 4-[2-(2-methoxy-4-nitrophenyl)-3-(4-nitrophenyl)tetrazol-2-ium-5-yl]benzene-1,3-disulfonate (WST-8), following the manufacturer's instructions. In this assay, WST-8 reacts with superoxide (·O_2_^-^) generated by xanthine oxidase (XO). The reaction produces a water-soluble formazan. A lower formazan signal indicates higher SOD activity. The SOD-like activity was therefore estimated from the WST-8 color signal. The WST-8/enzyme working solution and the reaction starting solution were prepared as instructed. The WST-8/enzyme working solution was mixed with the reaction starting solution and the SOD detection buffer. PBs (100 μg/mL), AS@PBs (100 μg/mL), or AS@PB-CTPs (100 μg/mL) were added. The mixture was incubated at 37 °C for 30 min. The absorbance was measured at 450 nm. The SOD-like activity was reported as the inhibition percentage.

CAT-like activity was evaluated by monitoring H_2_O_2_ breakdown using a commercial CAT assay kit. The kit measures the decrease in absorbance at 240 nm when H_2_O_2_ is converted to H_2_O and O_2_. PBs (100 μg/mL), AS@PBs (100 μg/mL), and AS@PB-CTPs (100 μg/mL) were mixed with the H_2_O_2_ substrate. The absorbance at 240 nm was recorded at different time points.

POD-like activity was evaluated by monitoring the oxidation of 3,3′,5,5′-tetramethylbenzidine (TMB) in the presence of H_2_O_2_. TMB was used as a color substrate. The solution turns blue after oxidation (TMB-ox). In a standard assay, TMB solution and H_2_O_2_ solution were mixed in a cuvette. Deionized water (ddH_2_O) was added to reach a total volume of 2 mL. AS@PB-CTPs (25, 50, 100, and 200 μg/mL) were added to start the reaction. The mixture was kept at room temperature for 15 min. The absorbance at 652 nm was recorded.

### 5.6 Cell lines and culture

HL-1 cardiomyocytes, L929 fibroblast-like cells and RAW 264.7 macrophages were purchased from the American Type Culture Collection (ATCC). HL-1 cells and L929 fibroblast-like cells were cultured in complete medium with 10% fetal bovine serum (FBS, Gibco) and 1% penicillin–streptomycin (Thermo Fisher Scientific). RAW 264.7 cells were cultured in medium with 10% FBS and no antibiotics. All cells were kept at 37 °C in a humidified incubator with 5% CO_2_. All cell lines were authenticated by short tandem repeat profiling and were confirmed to be mycoplasma-free before use. Cell numbers were counted with an automated cell counter (Cellometer Auto 1000, Nexcelom Bioscience). Trypan blue staining was used before counting. PD100 chambers were used for the counting step.

### 5.7 Macrophage polarization assay

Macrophage polarization markers were measured by flow cytometry. RAW 264.7 cells were seeded in 12-well plates at 1 × 10^5^ cells per well. LPS (300 ng/mL) was added for 6 h to activate the cells. Unstimulated cells were used as the control. After LPS treatment, the cells were treated for 4 h in serum-free medium with PBS, PB, free AS-IV, AS@PB, or AS@PB-CTP. The medium was then changed to complete medium with 10% FBS. The cells were incubated for another 20 h. The cells were collected and kept on ice. An anti-mouse CD16/32 antibody was added for 10 min to block non-specific binding. The cells were then stained on ice for 30 min with an antibody mix (1%) containing APC anti-F4/80 and PE-Cy7 anti-CD86. The cells were fixed and permeabilized. The cells were then stained at room temperature for 30 min with PB450 anti-CD206. The cells were finally resuspended in 300 μL PBS. The samples were analyzed on a Beckman Coulter flow cytometer (Life Science, USA).

### 5.8 Intracellular ROS measurement

In HL-1 cardiomyocytes, 2′,7′-dichlorodihydrofluorescein diacetate (DCFH-DA) was used to measure intracellular oxidative stress after treatment with PB, free AS-IV, AS@PB, or AS@PB-CTP. HL-1 cells (2 × 10^5^) were seeded in 12-well plates and incubated overnight. The cells were treated with PBS, PB, free AS-IV, AS@PB, or AS@PB-CTP for 8 h. The medium was then replaced with fresh medium. H_2_O_2_ (650 μM) was added, and the cells were incubated for another 24 h. The cells were collected and stained with DCFH-DA (10 μM) for 30 min. The cells were then observed by a fluorescence microscope. The cells were also analyzed by flow cytometry. All flow cytometry data were analyzed with FlowJo.

### 5.9 Apoptosis detection

Apoptosis was measured by annexin V-FITC and propidium iodide (PI) double staining using an apoptosis kit. HL-1 cells (2 × 10^5^) were treated according to the groups described above. The medium was then replaced with fresh medium. The cells were stained with annexin V-FITC and PI for 15 min at room temperature. The cells were then analyzed by flow cytometry.

### 5.10 Cellular uptake and cardiomyocyte-preferential internalization

For time-dependent cellular uptake analysis, HL-1 cardiomyocytes were seeded onto confocal culture dishes and cultured overnight. The cells were then incubated with fluorescently labeled AS@PB-CTP at a concentration of 100 μg/mL for 0, 2, 4, and 8 h. After incubation, the cells were washed three times with PBS to remove non-internalized nanoparticles, fixed with 4% paraformaldehyde, and counterstained with DAPI. Cellular uptake was visualized using confocal laser-scanning microscopy. DAPI and nanoparticle-associated fluorescence were detected using the corresponding excitation and emission channels. To evaluate CTP-mediated cellular selectivity, HL-1 cardiomyocytes and L929 fibroblast-like cells were seeded separately and incubated with fluorescently labeled AS@PB or AS@PB-CTP at an equivalent nanoparticle and fluorophore concentration of 100 μg/mL for 4h. Untreated cells were used as the negative control. After incubation, the cells were washed three times with cold PBS, detached, collected by centrifugation, and resuspended in PBS for flow-cytometric analysis. Debris and cell aggregates were excluded based on forward- and side-scatter characteristics, and nanoparticle uptake was evaluated according to the percentage of fluorescence-positive cells and mean fluorescence intensity.

### 5.11 The anti-PD-1/cTnI myocarditis model and treatment

Male BALB/c mice aged 8 weeks were purchased and randomly assigned to six groups: (i) Healthy Control, (ii) cTnI + anti-PD-1 + PBS, (iii) cTnI + anti-PD-1 + AS-IV, (iv) cTnI + anti-PD-1 + PB, (v) cTnI + anti-PD-1 + AS@PB, and (vi) cTnI + anti-PD-1 + AS@PB-CTP. All animal experiments were approved by the Institutional Animal Care and Use Committee and were performed in accordance with the ARRIVE guidelines.

Autoimmune myocarditis was induced in groups (ii)–(vi) by subcutaneous immunization with 0.1 mL of an emulsion containing 0.25 mg murine cardiac troponin I (cTnI) peptide and complete Freund's adjuvant on Days 1 and 7. The first immunization was used to initiate cardiac autoantigen sensitization, whereas the second immunization served as a booster to reinforce and synchronize the autoreactive immune response. Mice in the Healthy Control group received equal volumes of normal saline on the same schedule. To establish the anti-PD-1/cTnI myocarditis model, mice in groups (ii)–(vi) additionally received intraperitoneal injections of anti-PD-1 monoclonal antibody at 5 mg/kg every 48 h beginning on Day 7, for a total of five administrations. Mice in the Healthy Control group received vehicle injections according to the same regimen. The repeated anti-PD-1 regimen was used to sustain checkpoint blockade during ongoing cardiac autoantigen stimulation.

For treatment intervention, mice in group (ii) received PBS, whereas mice in groups (iii), (iv), (v), and (vi) received AS-IV, PB, AS@PB, or AS@PB-CTP, respectively. AS-IV was administered at 0.2 mg/kg, and PB, AS@PB, or AS@PB-CTP was administered at an equivalent nanoparticle dose of 15 mg/kg. Treatments were administered intravenously on Days 7, 10, 13, 16, and 19, for a total of five administrations. The first treatment on Day 7 coincided with the booster cTnI immunization and the initiation of anti-PD-1 blockade.

Throughout the experimental period, mice were monitored for general condition, survival, and body weight. On Day 21, transthoracic echocardiography was performed using a VisualSonics Vevo 2100 imaging system under light anesthesia to evaluate cardiac function, including left ventricular ejection fraction (EF) and fractional shortening (FS). Immediately after echocardiographic assessment, mice were euthanized, and blood and tissue samples were collected. Myocardial injury and inflammatory infiltration were evaluated by hematoxylin and eosin (H&E) staining of paraffin-embedded heart sections, and interstitial fibrosis was assessed by Masson's trichrome staining. Serum levels of CK-MB, TNF-α, and IL-6 were measured as biochemical indicators of myocardial injury and systemic inflammation. Major organs were collected for histological, molecular, and immune-related analyses as indicated.

To evaluate the biodistribution and cardiac accumulation of the nanoparticles, free ICG, ICG-labeled AS@PB, or ICG-labeled AS@PB-CTP was intravenously administered to mice via the tail vein. At predetermined time points after injection (0, 4, 8, 12, 24, and 48 h), mice were euthanized, and the major organs, including the heart, liver, spleen, lungs, and kidneys, were collected and imaged ex vivo using an IVIS Spectrum imaging system (PerkinElmer, USA). Fluorescence images were acquired using identical excitation/emission settings, exposure time, binning, and field of view for all experimental groups and time points. Regions of interest were manually drawn around each organ using Living Image software, and the fluorescence intensity was expressed as radiant efficiency ([photons/s/cm^2^/sr]/[μW/cm^2^]). Quantitative comparisons of organ fluorescence were performed at representative time points, including 4 and 24 h after injection. All imaging and quantitative analyses were conducted using the same acquisition and analysis parameters.

### 5.12 Western blotting

Heart tissues from each group were lysed in RIPA buffer supplemented with phosphatase inhibitors (Solarbio, Beijing, China) and protease inhibitors (Solarbio, Beijing, China). Protein concentration was measured using a BCA protein assay kit (Solarbio, Beijing, China). Proteins were analyzed by western blotting (WB) following a previously reported method. Band intensity was quantified using ImageJ.

### 5.13 Measurement of mRNA expression in heart tissues and cells

Selected PI3K–Akt-related genes (Ereg, Itga10, Thbs1, Spp1, Nr4a1, Fgfr2) were quantified via qRT-PCR using SYBR Green Master Mix (Vazyme, China). Total RNA was extracted from the heart from different groups using TRIzol™ reagent (Invitrogen, USA), following the manufacturer's instructions. The isolated RNA was converted into cDNA using a reverse transcription kit (Vazyme, China). GAPDH was used as the internal control. Primer sequences are listed in Supplementary Table S3.

### 5.14 Flow cytometry analysis of splenic immune cells

Single-cell suspensions were prepared from mouse spleens. Spleens were gently mashed through a 70 μm strainer into ice-cold FACS buffer, followed by erythrocyte lysisand washes. Cell counts and viability were assessed by trypan blue. For intracellular cytokine/granule readouts, 2 × 10^6^ cells were plated (24-/96-well plates) and stimulated 12 h at 37 °C, 5% CO_2_ with PMA (50 ng mL^−1^) and ionomycin (1 μg mL^−1^) in the presence of brefeldin A/monensin (protein transport inhibitor cocktail) to retain cytokines. All subsequent steps were performed protected from light. Live/dead discrimination was performed first using Zombie Aqua fixable dyefor 15 min at RT in PBS. After Fc blocking (anti-CD16/32, 10 min, 4 °C), surface staining was carried out for 30 min at 4 °C with the following antibody panel (final 0.2–0.5 μg per 10^6^ cells unless otherwise stated): CD45-PerCP-Cy5.5, CD3-FITC, CD4-APC, and CD8-APC-Cy7. Cells were then washed and fixed/permeabilized using Cytofix/Cytoperm (BD) according to the manufacturer's protocol, followed by intracellular staining for IFN-γ-PE for 30 min at 4 °C. After final washes, cells were resuspended in FACS buffer for acquisition. The standard gating strategy was: singlets → live cells (Zombie Aqua^-^) → CD45^+^ leukocytes → CD3^+^ T cells → CD4^+^ or CD8^+^ subsets; within CD8^+^ cells, IFN-γ^+^ fractions were quantified. Percentages are reported relative to parent gates as indicated in figure legends.

### 5.15 Multiplex immunofluorescence staining

Multiplex immunofluorescence staining with tyramide signal amplification (TSA) was performed on paraffin-embedded mouse heart tissue sections to detect CD68, TIM4, and CCR2. After deparaffinization and rehydration, sections were subjected to heat-induced antigen retrieval in citrate buffer (pH 6.0). Endogenous peroxidase activity was blocked, and nonspecific binding was blocked with 5% BSA. Multiplex detection was achieved through three sequential TSA staining rounds. In each round, sections were incubated with primary antibodies against CD68 (1:200), TIM4 (1:100), or CCR2 (1:150), followed by incubation with HRP-conjugated secondary antibody and fluorophore-tyramide signal development using TYR-520, TYR-570, or TYR-690, respectively, for 10 min. After each TSA reaction, antibody complexes were removed using antibody stripping buffer for 10 min, while the covalently deposited fluorophore signals were retained. Nuclei were counterstained with DAPI. Whole-slide images were acquired using a TissueFAXS multispectral slide scanner (TissueGnostics) with identical acquisition settings across all experimental groups. The acquired signals were pseudocolored as follows: CD68 in green, TIM4 in red, CCR2 in yellow, and DAPI in blue. Representative myocardial regions were selected for image presentation.

### 5.16 Statistical analysis

All data are shown as mean ± standard deviation (SD). Each experiment was repeated at least three times. Statistical analysis was performed using GraphPad Prism 10.0 (GraphPad, USA). A Student's t-test was used for comparisons between two groups. One-way ANOVA was used for comparisons among more than two groups. Significance was defined as P < 0.05. We used the following symbols: *P < 0.05, **P < 0.01, and ***P < 0.001.

## Funding

This work was supported by Innovation Team of the First Affiliated Hospital of 10.13039/501100011827Guangxi Medical University (YYZS2024003); Young Leader Talent Training Program of Guangxi Medical University(202307).

## CRediT authorship contribution statement

**Jing Zhou:** Conceptualization, Data curation, Investigation, Methodology, Software, Writing – original draft. **Jiali Tang:** Data curation, Investigation, Software. **Zehao Huang:** Data curation, Investigation, Visualization. **Zixuan Liang:** Data curation, Investigation. **Yunxi Huang:** Investigation, Visualization. **Qi Zeng:** Data curation, Investigation. **Chao Yu:** Data curation, Investigation. **Junjie Liu:** Data curation, Funding acquisition, Investigation. **Yuanyuan Chen:** Data curation, Investigation. **Sida Wang:** Conceptualization, Data curation, Investigation. **Nuo Yang:** Data curation, Funding acquisition, Investigation. **Yan Deng:** Conceptualization, Data curation, Funding acquisition, Methodology, Resources, Supervision, Writing – review & editing.

## Declaration of competing interest

The authors declare that they have no known competing financial interests or personal relationships that could have appeared to influence the work reported in this paper.

## Data Availability

Data will be made available on request.
