# Supplementary material for: Cardiac-targeting peptide–modified Prussian blue nanozymes loaded with Astragaloside IV for efficient ICI-myocarditis therapy
Source: Mater Today Bio. 2026 Jul 22;39:103482. doi: 10.1016/j.mtbio.2026.103482 (PMC13430262; doi:10.1016/j.mtbio.2026.103482)
Supplement: Multimedia component 1 [file mmc1.docx]

**Supplementary material**

**
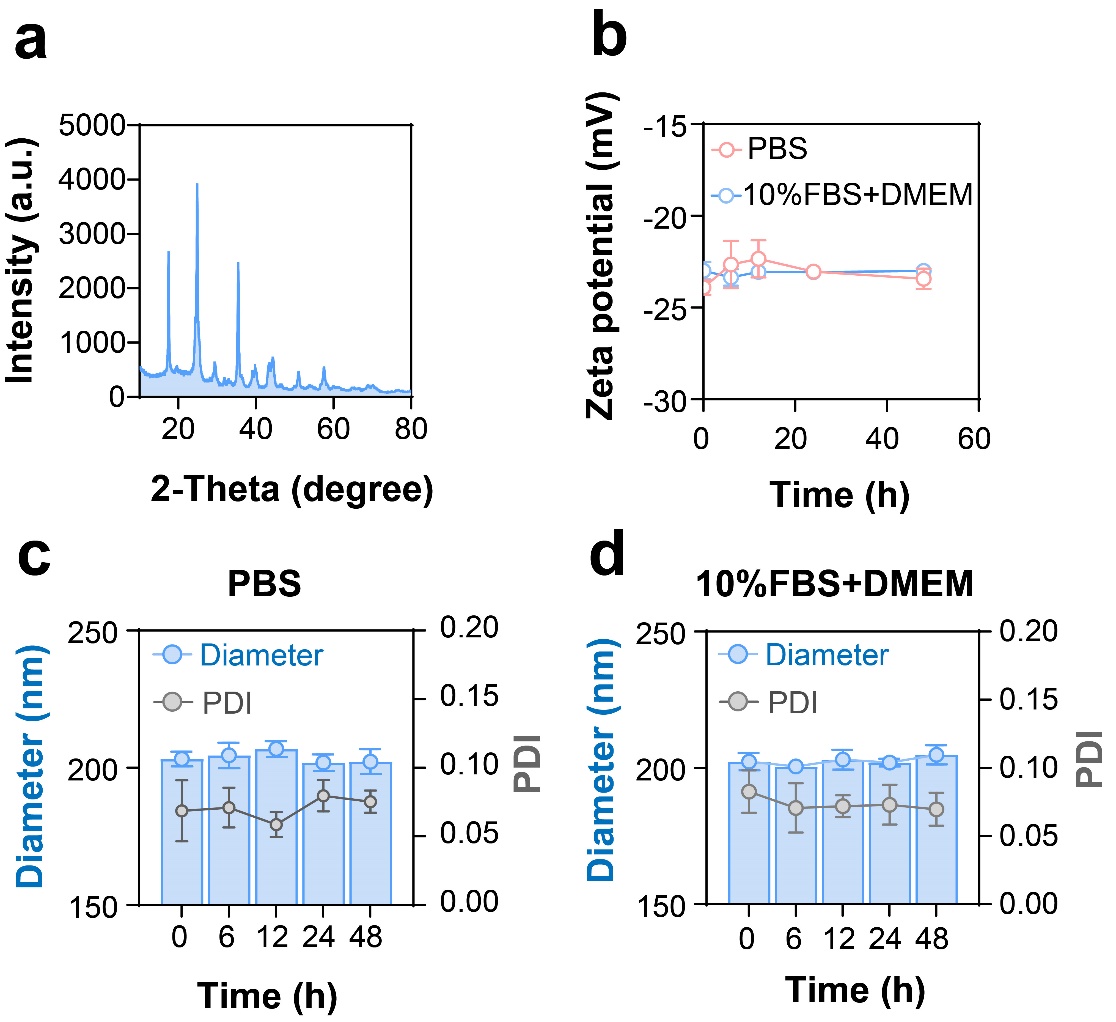
**

**Figure S1. Structural characterization and colloidal stability of AS@PB-CTP.**(a) X-ray diffraction (XRD) pattern of AS@PB-CTP. (b) Time-dependent changes in the zeta potential of AS@PB-CTP dispersed in PBS and DMEM supplemented with 10% FBS during incubation at 37 °C. (c, d) Time-dependent changes in the hydrodynamic diameter and polydispersity index (PDI) of AS@PB-CTP dispersed in PBS (c) and DMEM supplemented with 10% FBS (d) during incubation at 37 °C. Data are presented as mean ± SD (n=3).


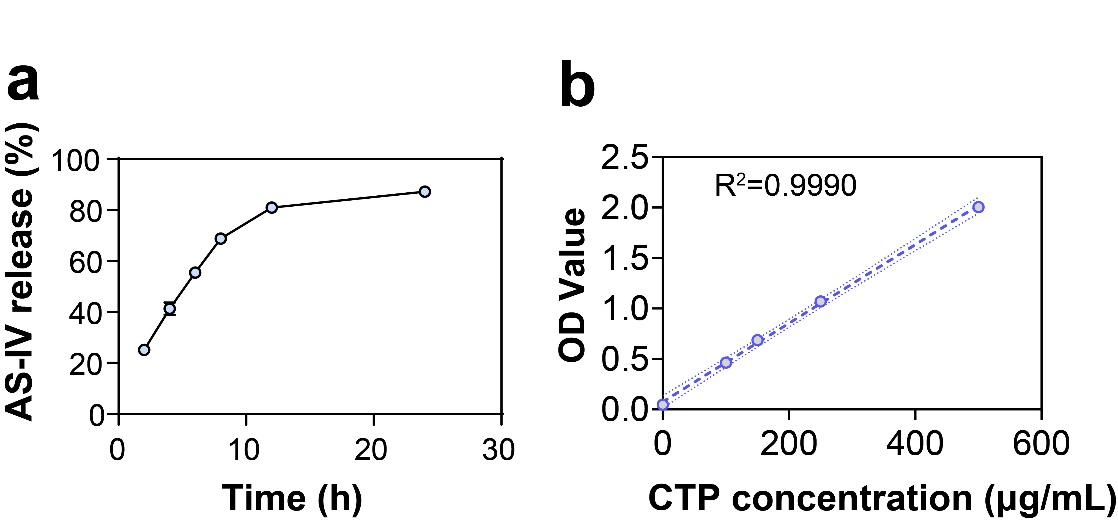


**Figure S2. In vitro AS-IV release profile and CTP calibration curve.**Cumulative release profile of AS-IV from AS@PB-CTP in PBS containing 0.5% polysorbate 80 over 24 h.

**
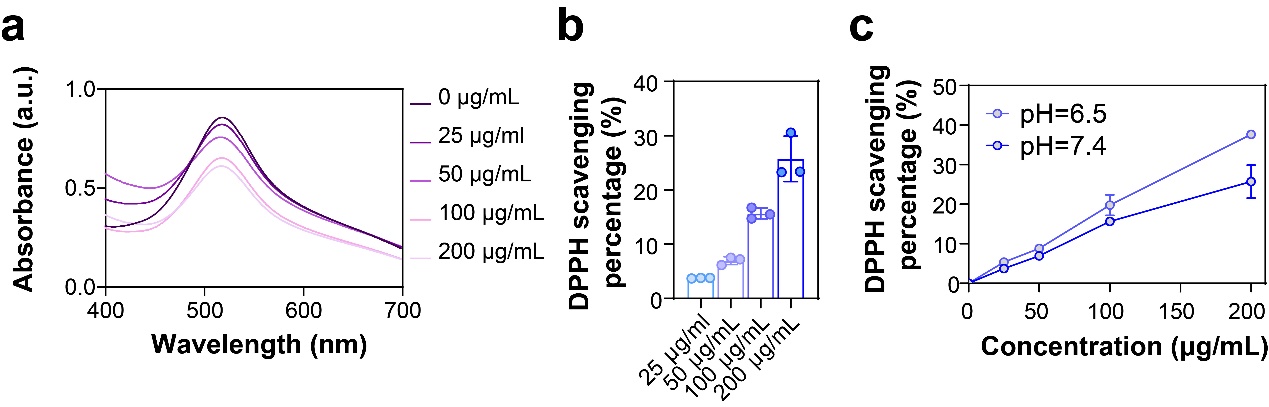
**

**Figure S3. DPPH radical-scavenging activity of AS@PB-CTP under different concentrations and pH conditions.**
(a) UV–vis absorption spectra of DPPH after incubation with AS@PB-CTP at concentrations of 0, 25, 50, 100, and 200 μg/mL. (b) Quantitative analysis of the DPPH radical-scavenging efficiency of AS@PB-CTP at different concentrations. (c) Comparison of DPPH radical-scavenging efficiency of AS@PB-CTP at pH 6.5 and pH 7.4. Data are presented as mean ± SD (n = 3).


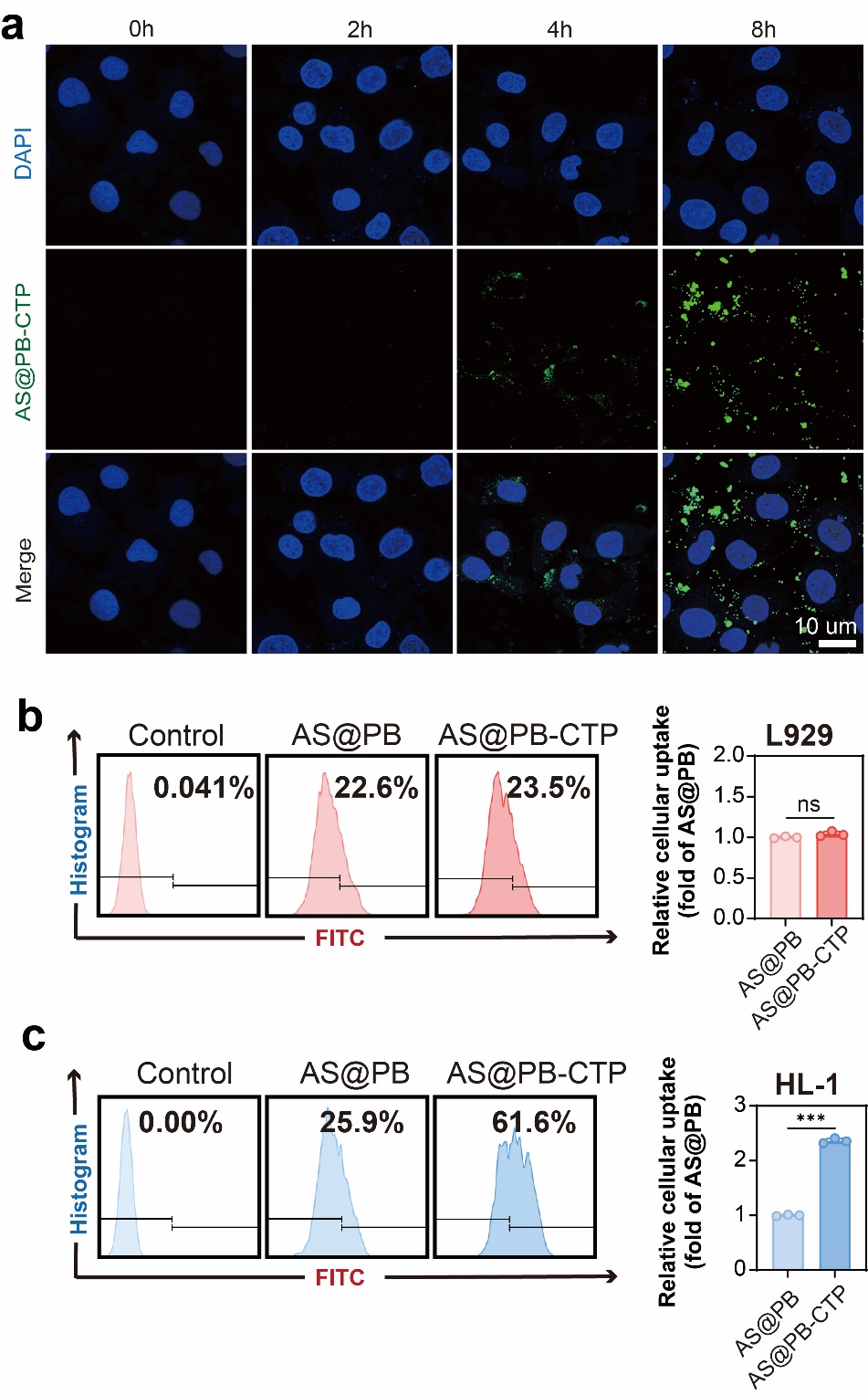


**Figure S4. Time-dependent uptake and CTP-mediated preferential cardiomyocyte uptake of FITC-labeled AS@PB-CTP.**(a) Representative confocal laser-scanning microscopy images showing the time-dependent internalization of FITC-labeled AS@PB-CTP in HL-1 cardiomyocytes after incubation for 0, 2, 4, and 8 h. Cell nuclei were stained with DAPI (blue), and FITC-labeled AS@PB-CTP is shown in green. Scale bar, 10 μm. (b, c) Representative flow-cytometric histograms and quantitative analysis of cellular uptake of FITC-labeled AS@PB and AS@PB-CTP in L929 mouse fibroblast-like cells (b) and HL-1 cardiomyocytes (c). Untreated cells were used as the control. The percentages shown in the histograms indicate FITC-positive cells. Cellular uptake was quantified based on the mean fluorescence intensity in the FITC channel and normalized to the corresponding AS@PB group. CTP modification did not significantly alter nanoparticle uptake by L929 cells, whereas it markedly enhanced uptake by HL-1 cardiomyocytes. Data are presented as mean ± SD from three independent experiments. ns, not significant; ****P* < 0.001.


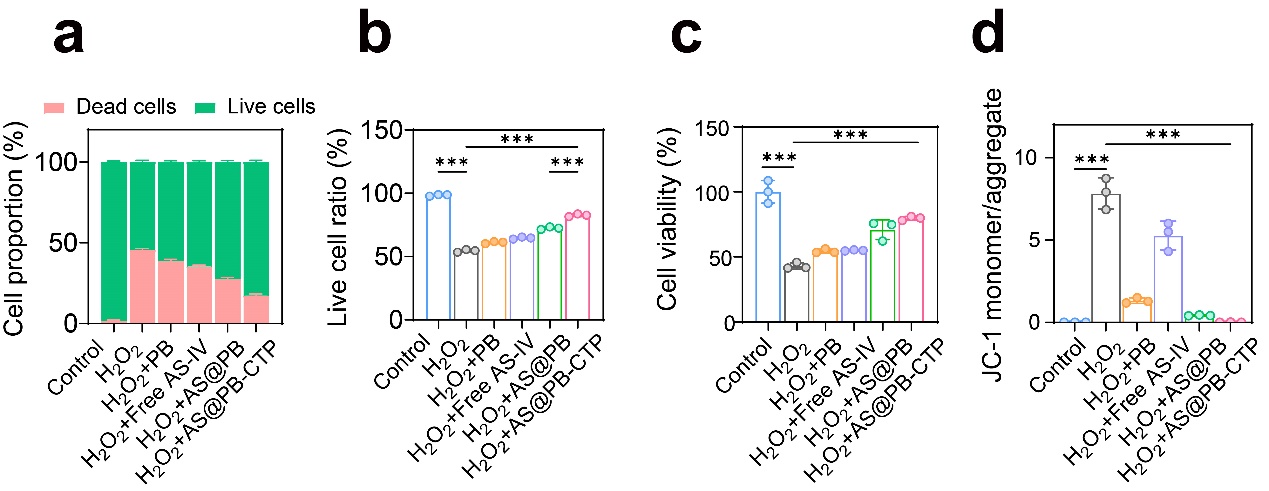


**Figure S5. Cytoprotective effects of AS@PB-CTP against H₂O₂-induced oxidative injury in HL-1 cardiomyocytes.**(a, b) Quantitative analysis of live/dead staining in HL-1 cells following the indicated treatments, including the proportions of live and dead cells (a) and the relative live cell ratio (b). (c) Cell viability of H₂O₂-stimulated HL-1 cells treated with PB, free AS-IV, AS@PB, or AS@PB-CTP, as determined using the CCK-8 assay. (d) Quantitative analysis of the JC-1 monomer-to-aggregate fluorescence ratio, reflecting mitochondrial membrane potential changes in HL-1 cells after the indicated treatments. Data are presented as mean ± SD from three independent experiments. ****P* < 0.001; statistical comparisons are indicated by the connecting lines.


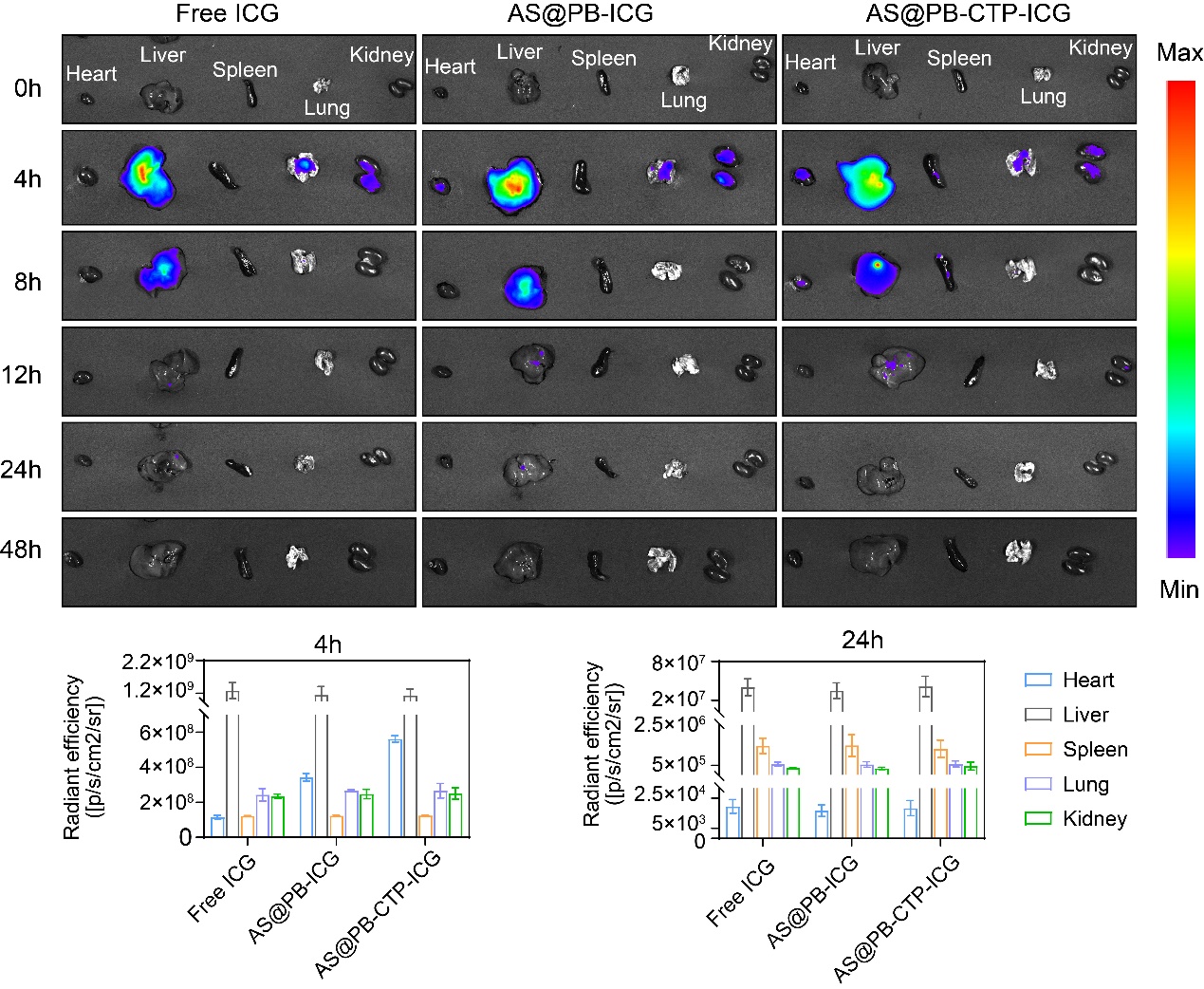


**Figure S6. Time-dependent ex vivo biodistribution of free ICG, AS@PB-ICG, and AS@PB-CTP-ICG.**

Representative ex vivo fluorescence images of major organs collected at 0, 4, 8, 12, 24, and 48 h after intravenous injection of free ICG, AS@PB-ICG, or AS@PB-CTP-ICG. Organs are arranged from left to right as heart, liver, spleen, lung, and kidney. Quantitative radiant-efficiency analyses of the heart, liver, spleen, lung, and kidney at 4 and 24 h are shown below the fluorescence images. Data are presented as mean ± SD (n = 3).


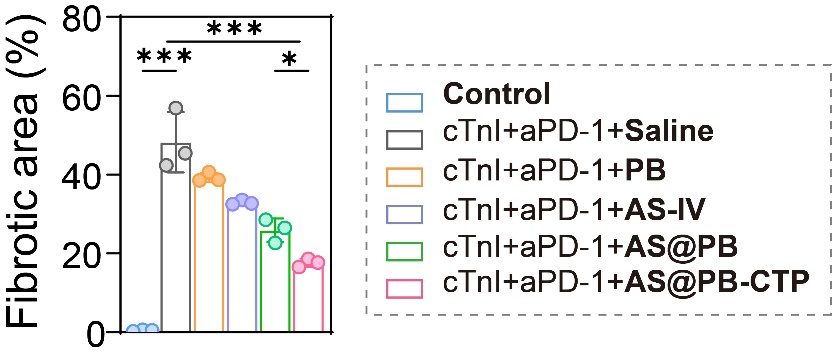


**Figure S7. Quantitative analysis of myocardial fibrosis following the indicated treatments.**
The collagen-positive fibrotic area in Masson’s trichrome-stained cardiac sections was quantified and expressed as a percentage of the total myocardial tissue area. Data are presented as mean ± SD (n = 3). *P < 0.05 and ***P < 0.001; statistical comparisons are indicated by the connecting lines.


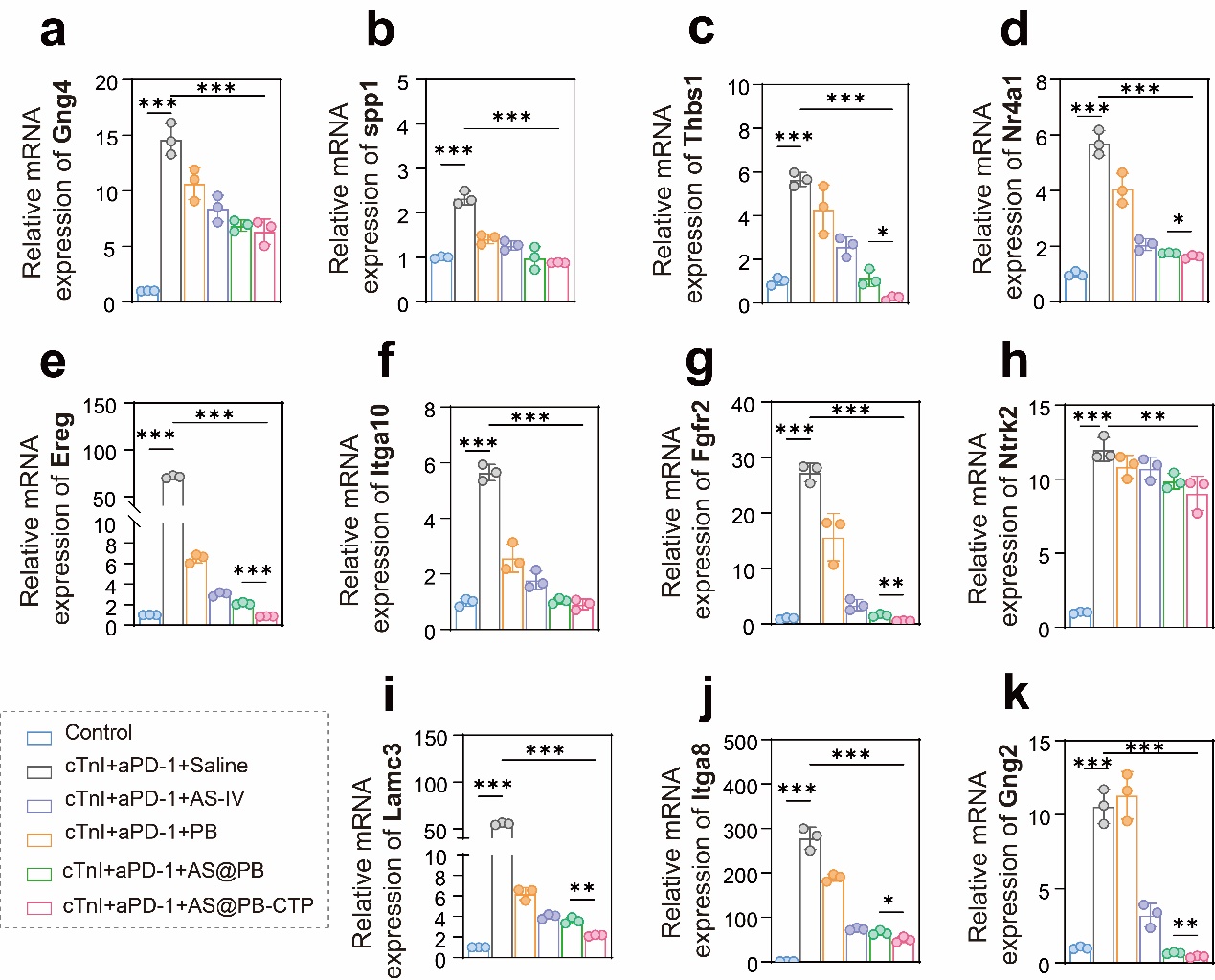


**Figure S8. AS@PB-CTP attenuates cardiac PI3K–Akt–associated transcriptional programs in the PD-1/TnI model of ICI-myocarditis.**

(a–k) Cardiac qRT–PCR showing that genes enriched in the PI3K–Akt pathway are elevated in the PBS (model) group and only partially reduced by Free AS-IV or PB alone, whereas AS@PB-CTP markedly downregulates *Gng4* (a), *Spp1* (b), *Thbs1* (c), *Nr4a1* (d), *Ereg* (e), *Itga10* (f), *Fgfr2* (g), *Ntrk2* (h), and remodeling-associated transcripts *Lamc3* (i), *Itga8* (j), and *Gng2* (k), in Control mice and cTnI+aPD-1–challenged mice treated with saline, AS-IV, PB, AS@PB, or AS@PB-CTP. Compared with the myocarditis (cTnI+aPD-1+Saline) group, **AS@PB-CTP reduced the induction of these transcripts**, indicating suppression of PI3K–Akt–linked pathogenic gene programs in the heart. Data are presented as mean ± SD (n = 3). One-way ANOVA with Tukey’s post hoc test was used, **P* < 0.05, ***P* < 0.01, and ****P* < 0.001; statistical comparisons are indicated by the connecting lines.


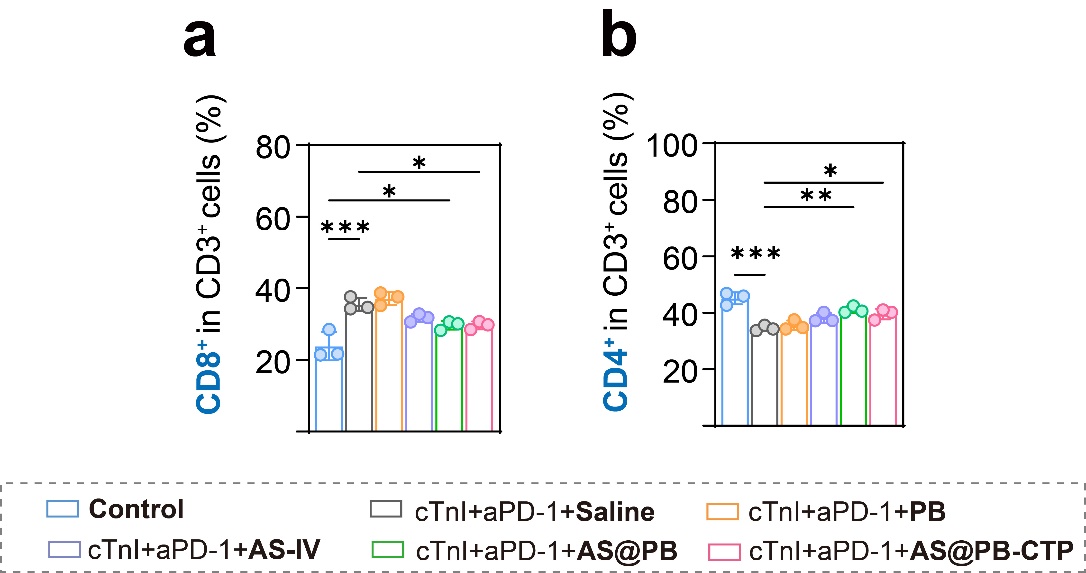


**Figure S9. Quantitative analysis of splenic T-cell subsets following the indicated treatments.**(a) Percentage of CD8⁺ T cells within the splenic CD3⁺ T-cell population. (b) Percentage of CD4⁺ T cells within the splenic CD3⁺ T-cell population. Data are presented as mean ± SD (n = 3). **P* < 0.05, ***P* < 0.01, and ****P* < 0.001; statistical comparisons are indicated by the connecting lines.

**
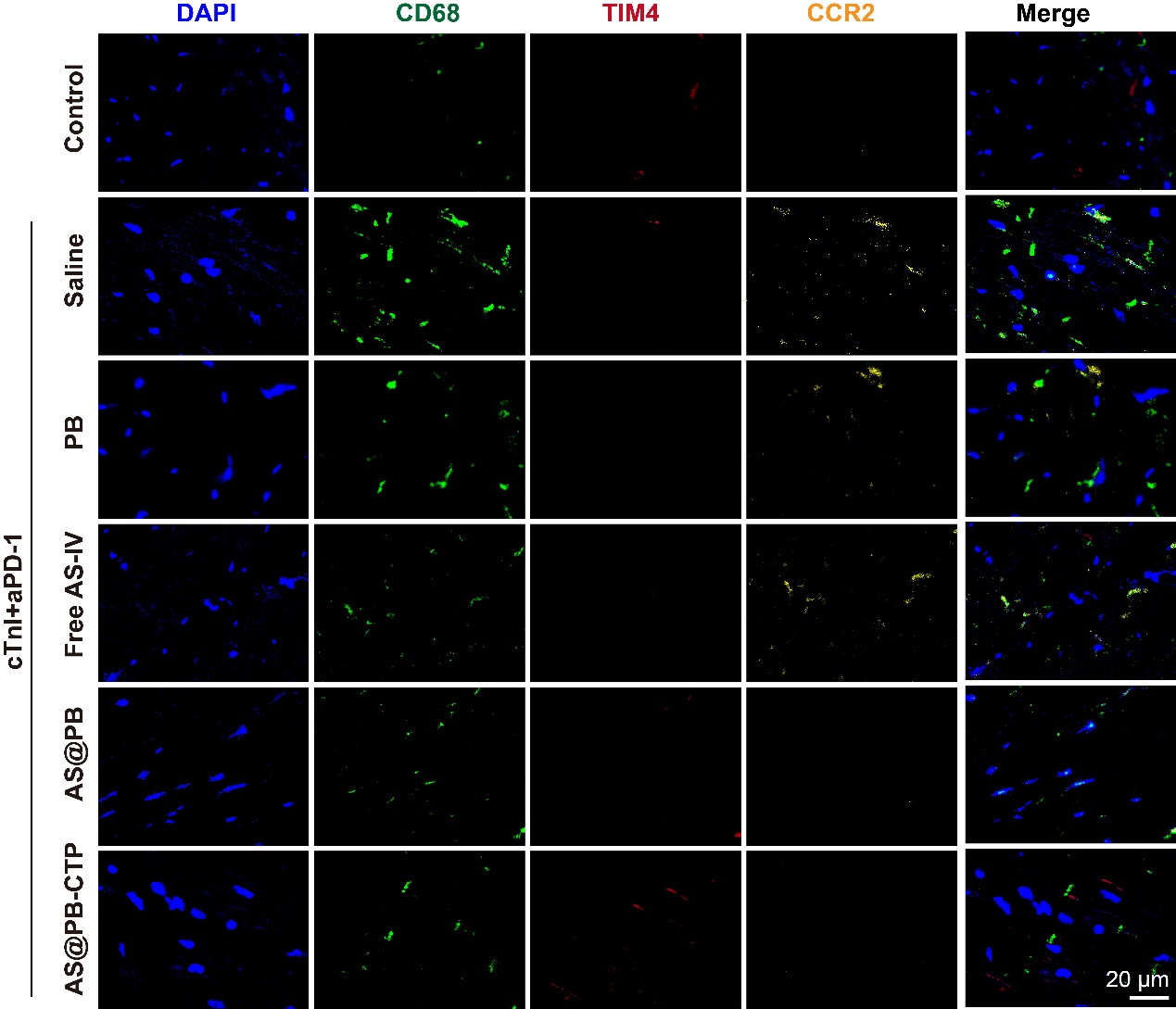
**

**Figure S10. Immunofluorescence characterization of resident-like and recruited macrophage populations in cardiac tissues.**

Representative immunofluorescence images of myocardial sections from the indicated groups stained with DAPI (blue), CD68 (green), TIM4 (red), and CCR2 (orange). CD68 was used as a pan-macrophage marker, whereas TIM4 and CCR2 were used as phenotypic markers associated with resident-like and recruited/infiltrating monocyte-derived macrophages, respectively. Merged images show the spatial distribution and colocalization of the indicated markers. Scale bar, 20 μm.


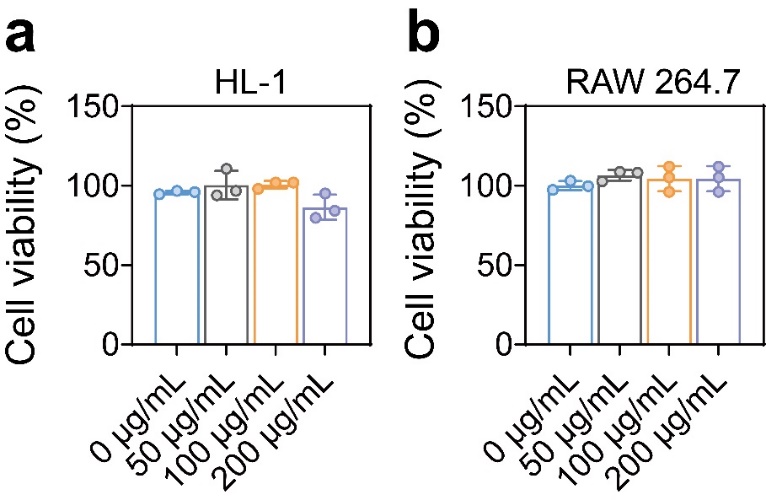


**Figure S11. Biocompatibility of AS@PB-CTP nanoparticles in vitro.**
(a) Cell viability of HL-1 cardiomyocytes after 24 h incubation with increasing concentrations (0, 50, 100, and 200 μg/mL) of AS@PB-CTP, as measured by CCK-8 assay. (b) Cell viability of RAW 264.7 macrophages treated under the same conditions. Data are presented as mean ± SD (n = 3).


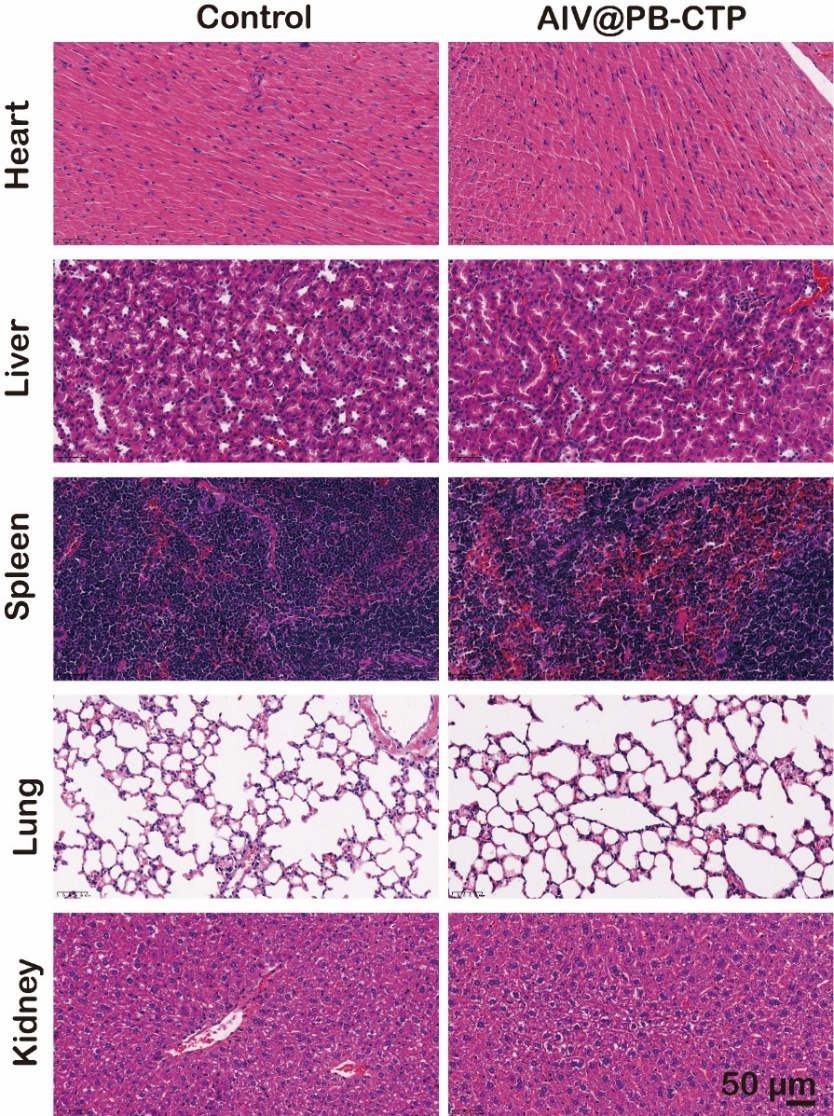


**Figure S12.** **Histological evaluation of major organs following AS@PB-CTP administration.**

Representative H&E-stained sections of the heart, liver, spleen, lung, and kidney from mice treated with PBS (Control) or AS@PB-CTP nanozymes. All major organs exhibited normal histoarchitecture with no apparent inflammatory infiltration, necrosis, or structural abnormalities in the AS@PB-CTP group compared to controls, indicating favorable in vivo biocompatibility and negligible systemic toxicity. Scale bar: 50 μm.


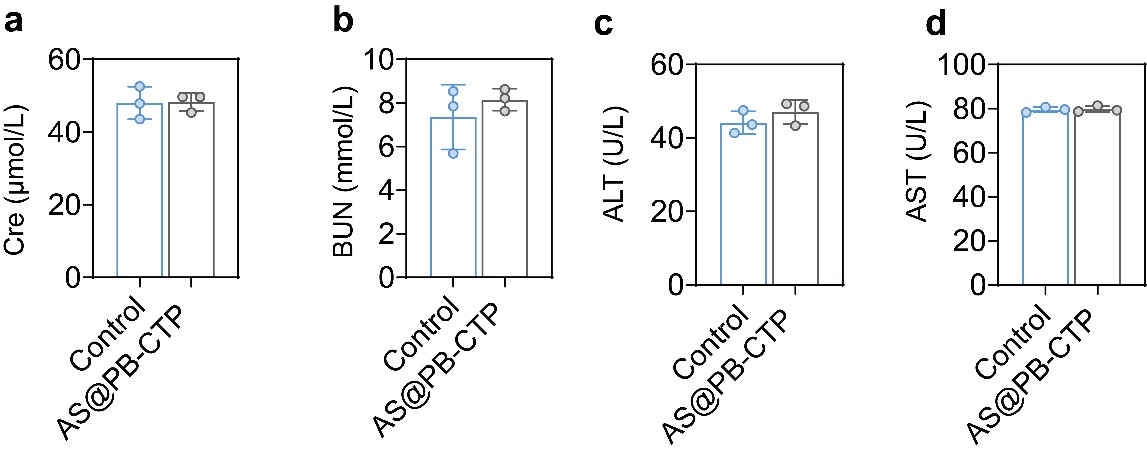


**Figure S13.** **Serum biochemistry analysis after systemic administration of AS@PB-CTP.**Levels of (a) creatinine (Cre), (b) blood urea nitrogen (BUN), (c) alanine aminotransferase (ALT), and (d) aspartate aminotransferase (AST) in mice treated with PBS or AS@PB-CTP (200 μg/mL, i.v., 24 h). No significant differences were observed between groups, confirming the absence of acute renal or hepatic toxicity. Data are presented as mean ± SD (n = 3).


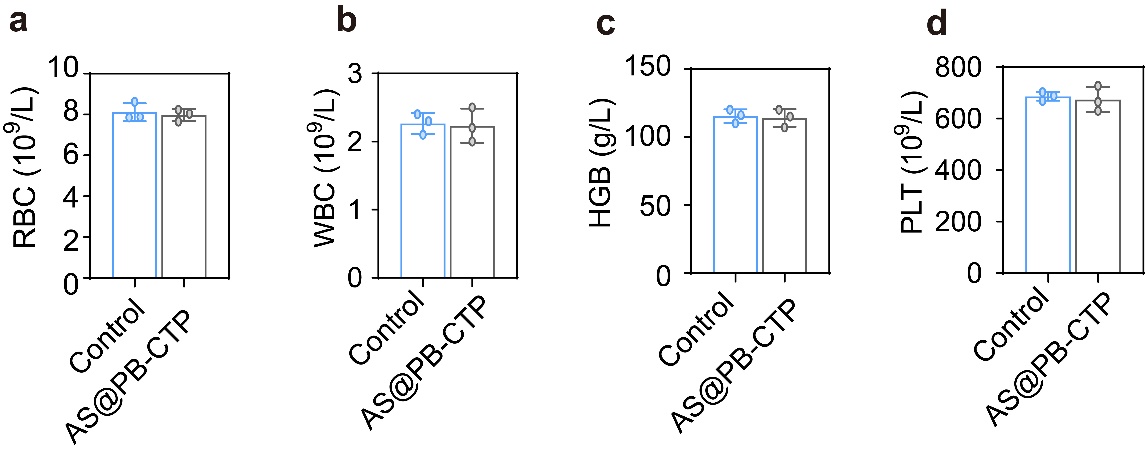


**Figure S14.** Red blood cells (RBC, a), white blood cells (WBC, b), hemoglobin (HGB, c), and platelets (PLT, d) in healthy mice after treatment with AS@PB-CTP compared with treated with PBS. Data are presented as mean ± SD (n = 3).


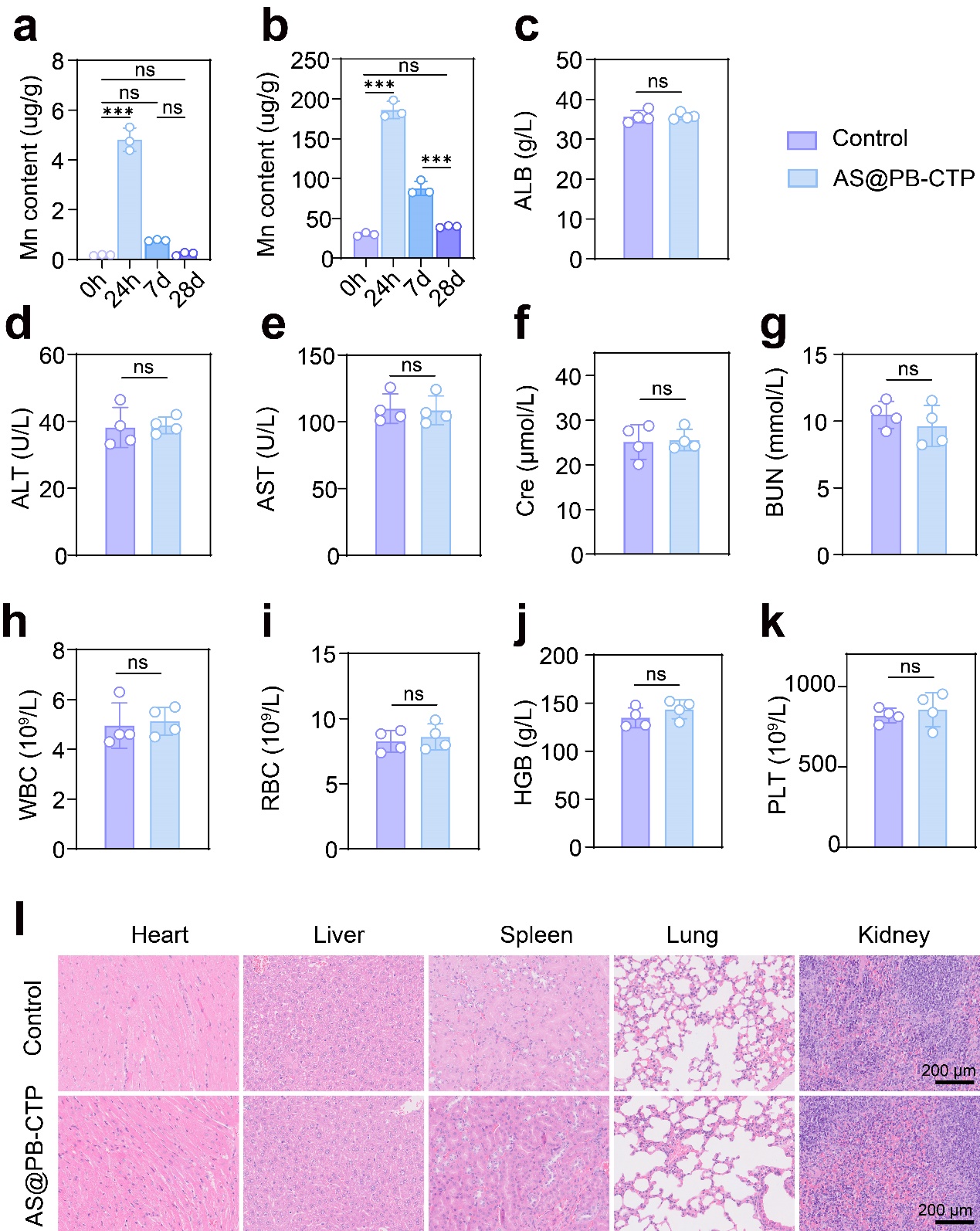


**Figure S15. Long-term in vivo fate and biosafety evaluation of AS@PB-CTP.**

(a) Time-dependent Mn content in cardiac tissue at 0 h, 24 h, 7 days, and 28 days after intravenous administration of AS@PB-CTP. (b) Time-dependent Mn concentration in blood at the indicated time points. (c–g) Serum biochemical parameters, including ALB, ALT, AST, creatinine, and BUN, measured on Day 28. (h–k) Hematological parameters, including WBC, RBC, HGB, and PLT, measured on Day 28. (l) Representative H&E-stained images of the heart, liver, spleen, lungs, and kidneys from the control and AS@PB-CTP groups on Day 28. Data are presented as mean ± SD. ***P < 0.001; ns, not significant. Scale bars, 200 μm.

**Supplementary Table S1.** Particle size; PDI; Encapsulation efficiency and Loading efficiency of AS@PB-CTP.

|  | Particle size  (nm) | PDI | Encapsulation efficiency (EE%) | Loading efficiency (LE%) |
| --- | --- | --- | --- | --- |
| 1 | 201.1 | 0.051 | 62.4 | 5.6 |
| 2 | 200.1 | 0.082 | 64.5 | 5.32 |
| 3 | 200.9 | 0.084 | 63.3 | 5.19 |
| Mean | 200.7 | 0.072 | 63.4 | 5.37 |
| SD | 0.53 | 0.019 | 1.05 | 0.21 |

**Supplementary Table S2.** Quantitative determination of CTP content in AS@PB-CTP.

|  | OD Value | Conjugated CTP (mg) | CTP loading content (wt%) |
| --- | --- | --- | --- |
| 1 | 1.74 | 4.13 | 17.10 |
| 2 | 1.89 | 3.78 | 15.64 |
| 3 | 1.87 | 3.84 | 15.87 |
| Mean |  | 3.92 | 16.20 |
| SD |  | 0.19 | 0.79 |

**Supplementary Table S3.** Primer information for mouse.

| Gene | Forward | Reverse |
| --- | --- | --- |
| *Ereg* | TTGGGTCTTGACGCTGCTTT | TGCATGATGGGATCACGGTTG |
| *Itga10* | AGGCCGAATTTGGATACAGTG | GAGCAACGATAAACATCCCCTC |
| *Gng4* | GCAGGGTCTTCTGTGGTCCG | TTCCTTCATTCCTGCACTCCC |
| *Spp1* | ATCTCACCATTCGGATGAGTCT | TGTAGGGACGATTGGAGTGAAA |
| *Thbs1* | CCTGCCAGGGAAGCAACAA | ACAGTCTATGTAGAGTTGAGCCC |
| *Nr4a1* | GAGTTCGGCAAGCCTACCAT | GTGTACCCGTCCATGAAGGTG |
| *Fgfr2* | GCTATAAGGTACGAAACCAGCAC | GGTTGATGGACCCGTATTCATTC |
| *Lamc3* | CAGAAAACCTATGGCCGTCCT | CCAAACGTGTTGAGCCGATCT |
| *Itga8* | TGTCTGGCGTTCAACTTGGAT | TCCAGTGAGTAGCCGAAGTAG |
| *Gng2* | ACCGCCAGCATAGCACAAG | AGTAGGCCATCAAGTCAGCAG |
| *Arg1* | TGTCCCTAATGACAGCTCCTT | GCATCCACCCAAATGACACAT |
| *CD206* | CTCTGTTCAGCTATTGGACGC | TGGCACTCCCAAACATAATTTGA |
| *YM-1* | TCAACGGTTTTTCCACAGTGC | TCCCAGCTGGTACAGCAGAC |
| *Fizz1* | CCTGCTGGGATGACTGCTAC | CAGTGGTCCAGTCAACGAGT |
